# Supplementary material for: Correcting tau isoform ratios with a long-acting antisense oligonucleotide alleviates 4R-tauopathy phenotypes
Source: Mol Ther Nucleic Acids. 2025 Mar 5;36(2):102503. doi: 10.1016/j.omtn.2025.102503 (PMC11979468; doi:10.1016/j.omtn.2025.102503)
Supplement: Document S1. Figures S1–S10 and Tables S1–S3 [file mmc1.pdf]

OMTN, Volume *36*

Supplemental information

Correcting tau isoform ratios with a long-acting antisense oligonucleotide alleviates 4R-tauopathy phenotypes

Kuniyuki Iwata-Endo, Kentaro Sahashi, Kaori Kawai, Yusuke Fujioka, Yohei Okada, Eri Watanabe, Nobuyuki Iwade, Minaka Ishibashi, Moniruzzaman Mohammad, Asraa Faris Aldoghachi, Dilina Tuerde, Tsuyoshi Fujiwara, Shinobu Hirai, Haruo Okado, Masahisa Katsuno, Hirohisa Watanabe, Kayoko Kanamitsu, Masahiro Neya, Shinsuke Ishigaki, and Gen Sobue

Supplemental Figure Legends

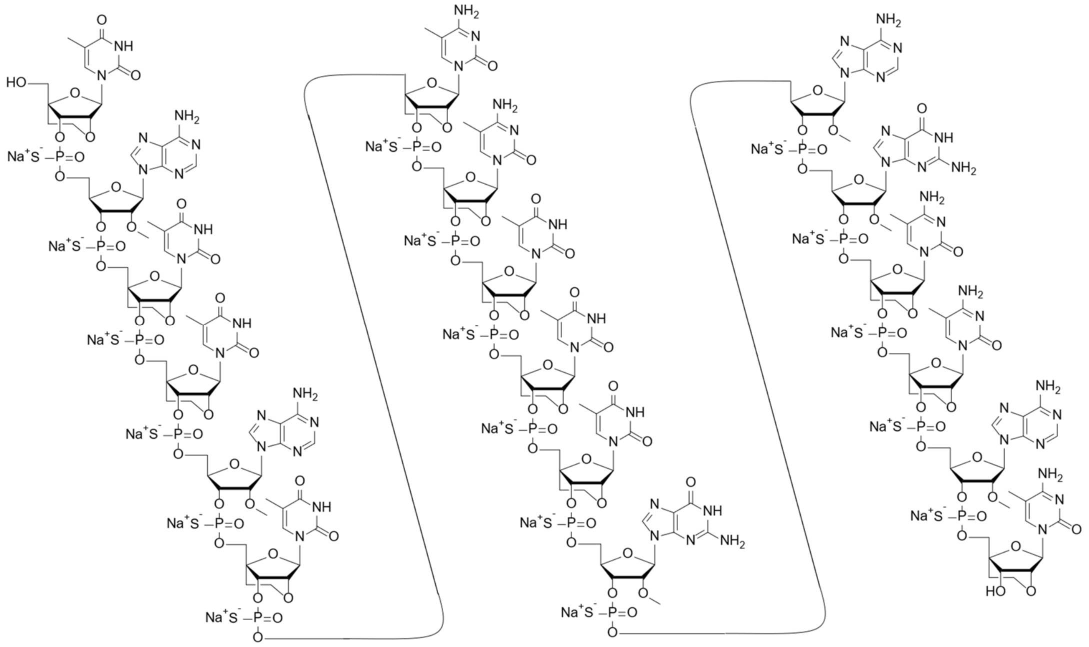

Figure S1. The structural formula of EN-06.

The structural formula of EN-06 sodium salt is shown.

Molecular Formula: $C_{210}H_{253}N_{59}O_{110}P_{17}S_{17}Na_{17}$

Molecular Weight: 6826.0370

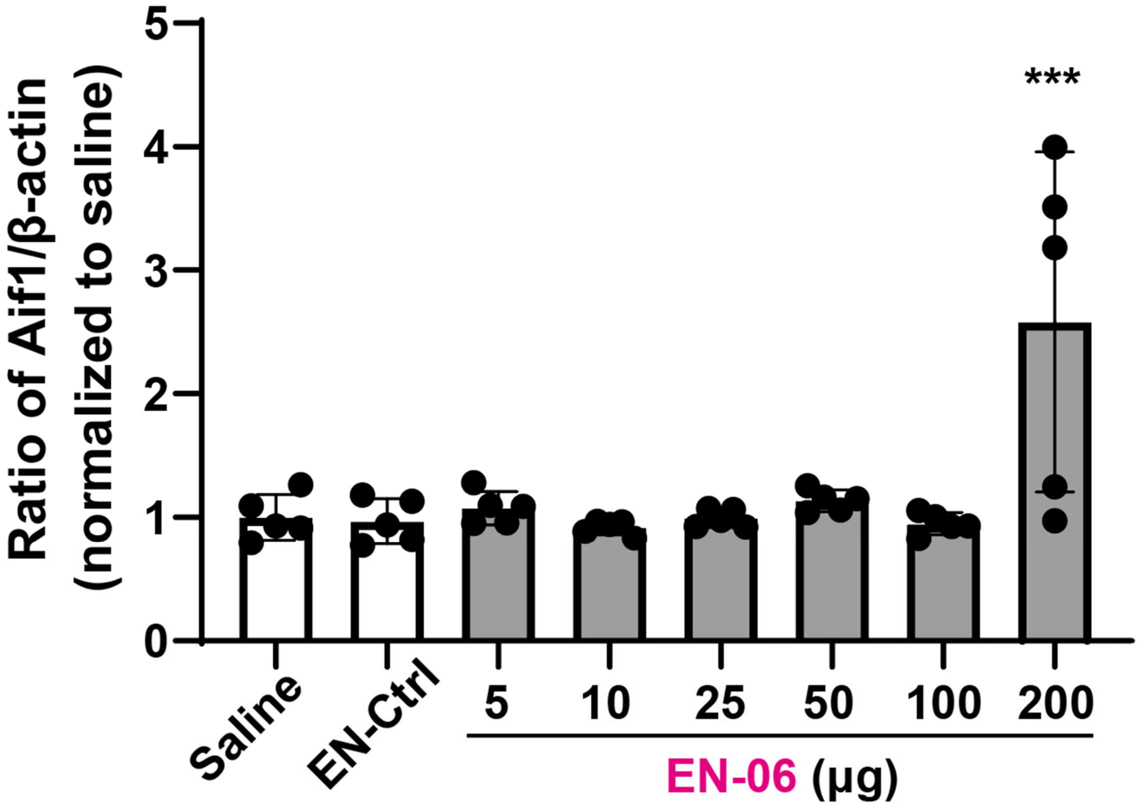

Figure S2. Inflammatory gene expression in the brain after a single ICV administration of EN-06.

The expression level of *Aif1*, which encodes the Iba1 protein, in brain homogenates from mice administered increasing doses (5, 10, 25, 50,100, and 200 μg) of EN-06 were assessed. The data shown represent the mean ± SD and are shown as *Aif1* per *β-actin* (n = 5 for each; one-way ANOVA; ***P < 0.001).

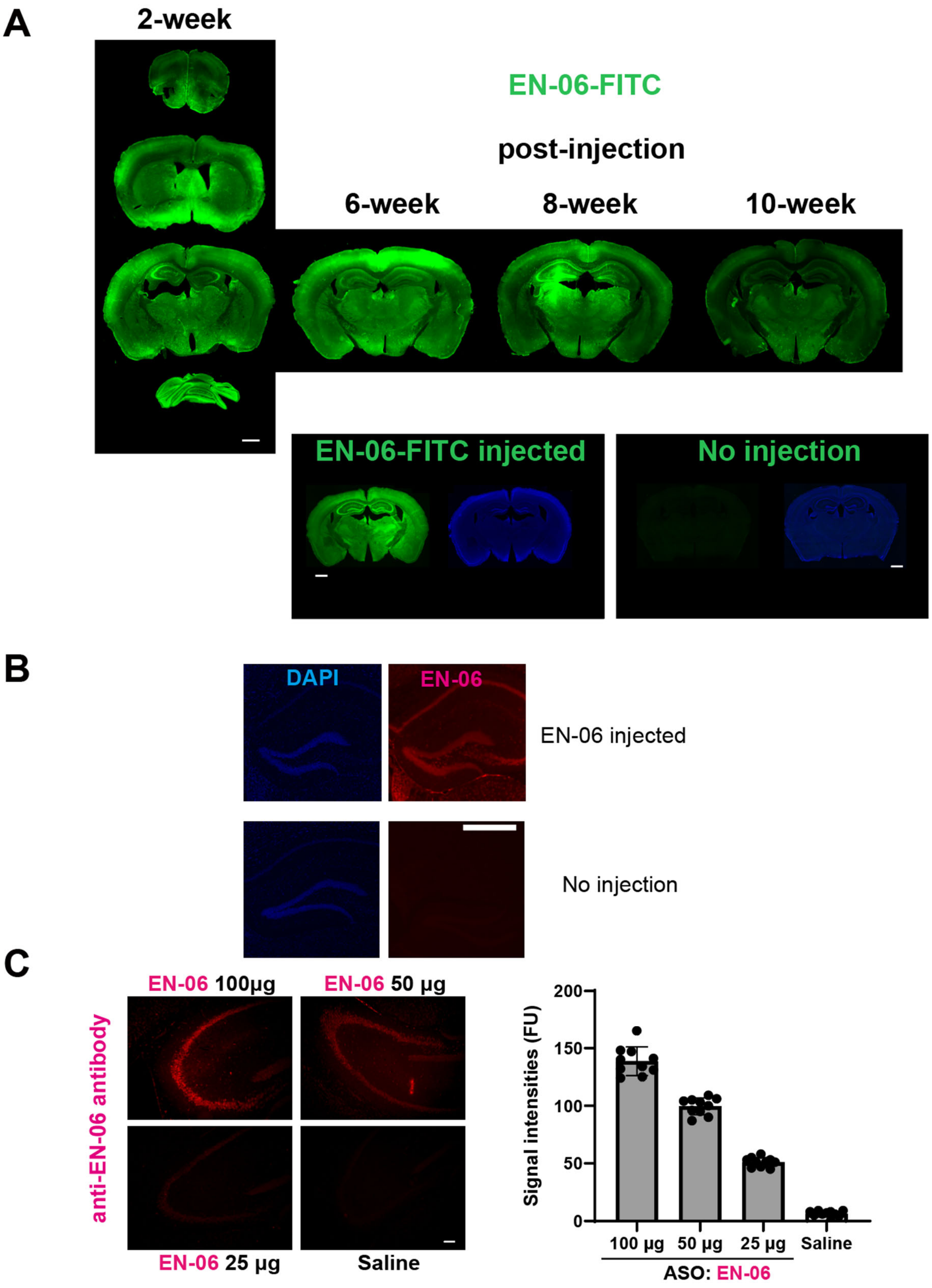
A
2-week
EN-06-FITC
post-injection
6-week
8-week
10-week
EN-06-FITC injected
No injection
B
DAPI
EN-06
EN-06 injected
No injection
C
EN-06 100μg
EN-06 50 μg
anti-EN-06 antibody
EN-06 25 μg
Saline
Signal intensities (FU)
200
150
100
50
0
100 μg
50 μg
25 μg
Saline
ASO: EN-06

Figure S3. Brain tissue distribution of ENA-ASOs targeting human *MAPT* exon 10.

(A) The brain distribution of FITC-labelled EN-06 at 2-, 6-, 8-, and 10 weeks post-ICV injection of 50 µg. A negative control section without injecting FITC-labelled EN-06 is shown with the section of FITC-labelled EN-06 at 6 weeks below. Scale bar = 1.0 mm. (B) Immunofluorescent study using a custom antibody against EN-06 in the brain. Mouse brain sections at 6 weeks post-ICV injection of 50 µg EN-06 were stained with an antibody against EN-06 (top). The negative control brain sections without EN-06 injection were stained with the same antibody (bottom). Scale bar = 0.5 mm. (C) Immunofluorescence imaging of hippocampal sections was done with a custom antibody against EN-06 after ICV injection of three different EN-06 doses (left panel). Scale bar = 100 µm. Signal intensities were quantified and plotted (right panel). The data shown represent the mean ± SD.

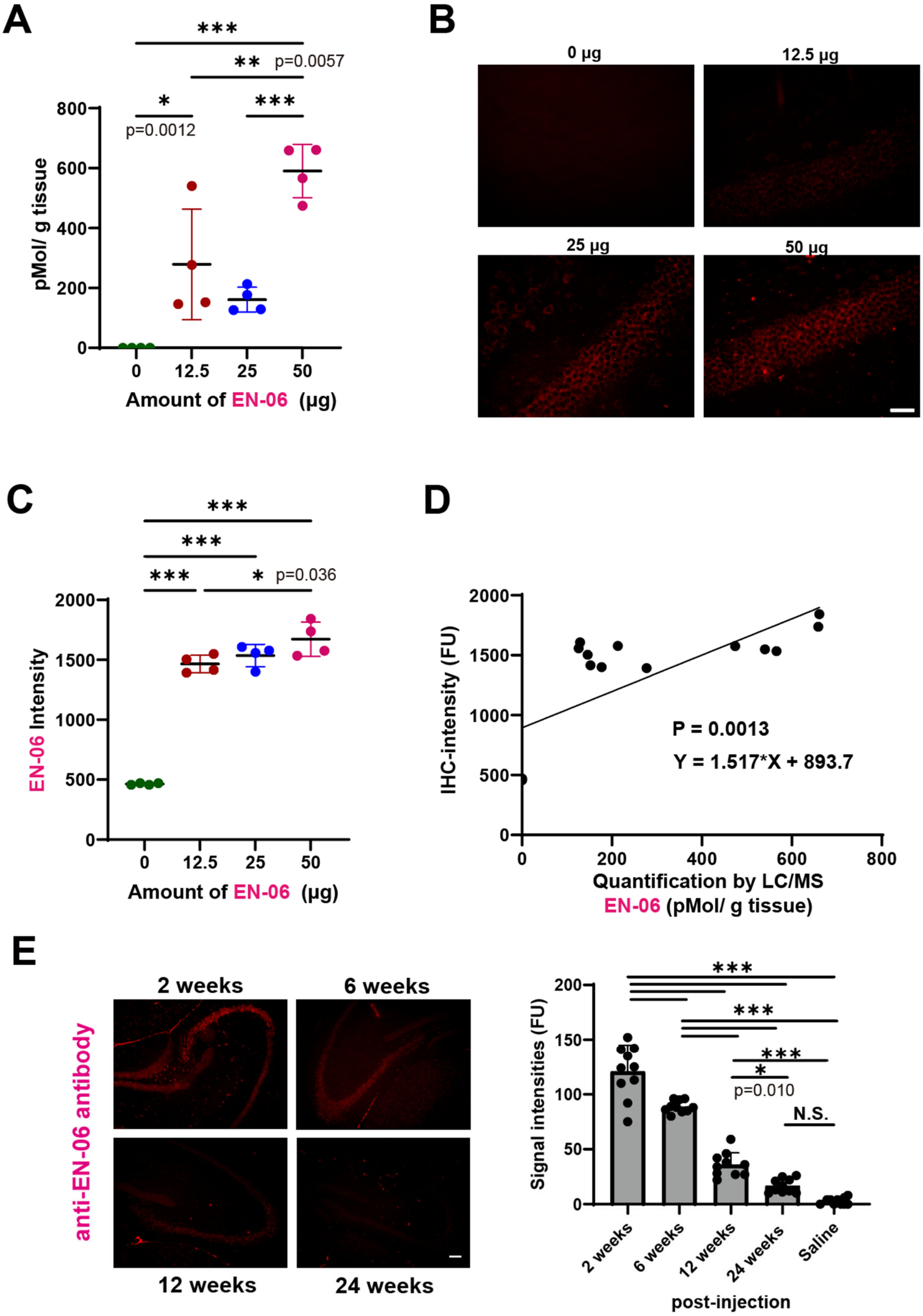
A
***
**
p=0.0057
*
***
p=0.0012
pMol/ g tissue
800
600
400
200
0
0
12.5
25
50
Amount of EN-06 (μg)
B
0 μg
12.5 μg
25 μg
50 μg
C
***
***
***
*
p=0.036
EN-06 Intensity
2000
1500
1000
500
0
0
12.5
25
50
Amount of EN-06 (μg)
D
IHC-intensity (FU)
2000
1500
1000
500
0
P = 0.0013
Y = 1.517*X + 893.7
0
200
400
600
800
Quantification by LC/MS
EN-06 (pMol/ g tissue)
E
anti-EN-06 antibody
2 weeks
6 weeks
12 weeks
24 weeks
Signal intensities (FU)
200
150
100
50
0
***
***
***
*
p=0.010
N.S.
2 weeks
6 weeks
12 weeks
24 weeks
Saline
post-injection

Figure S4. LC/MS-based and immunofluorescence-based quantification of EN-06 in the brain.

(A) The absolute tissue concentration of EN-06 was determined in the hippocampus of mice administered 4 doses (0, 12.5, 25, and 50 µg) of EN-06 by combining solid-phase extraction and LC/MS. The concentrations of EN-06 in the hippocampus of mice at 6 weeks post-injection were plotted and are shown as pMol/ g tissue (n = 4 for each dose; one-way ANOVA). (B) Immunofluorescent imaging was performed with a custom antibody against EN-06 using hippocampal sections of the mice shown in (A). Scale bar = 40 µm. (C) Signal intensities of the images in (B) were quantified and plotted. (D) Correlation of the absolute tissue concentrations of EN-06 as determined by LC/MS and immunofluorescence imaging ($P = 0.0013$). (E) Immunofluorescence imaging of hippocampal sections at various time points post-ICV injection of a 50-µg dose of EN-06 into 6-week-old hTau mice. Hippocampal sections were stained with a custom antibody against EN-06 (left panel). Scale bar = 100 µm. Signal intensities of the immunofluorescent images were quantified and plotted (right panel). $^{*}P < 0.05$, $^{**}P < 0.01$, $^{***}P < 0.001$.

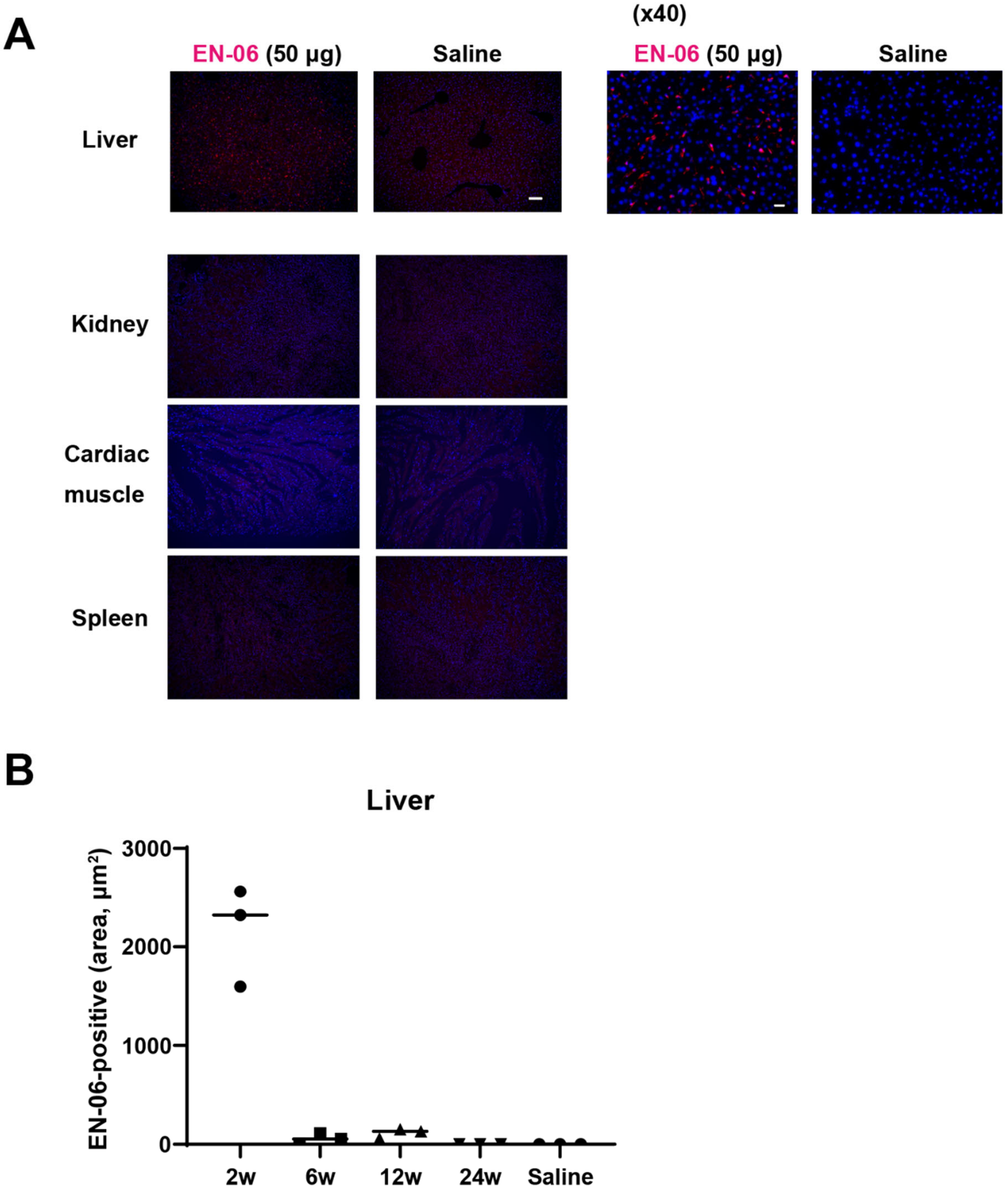

Figure S5. General tissue distribution of EN-06. (A) Immunofluorescent images of liver, kidney, cardiac muscle, and spleen sections of mice ICV injected 50 µg of EN-06 or saline. Sections were stained with a custom antibody against EN-06. Scale bar = 100 µm for left

images, 20 μm for right images. (B) The signal intensities in the liver sections were quantified and plotted (right panel). The data shown represent the mean ± SD (n = 3 for each).

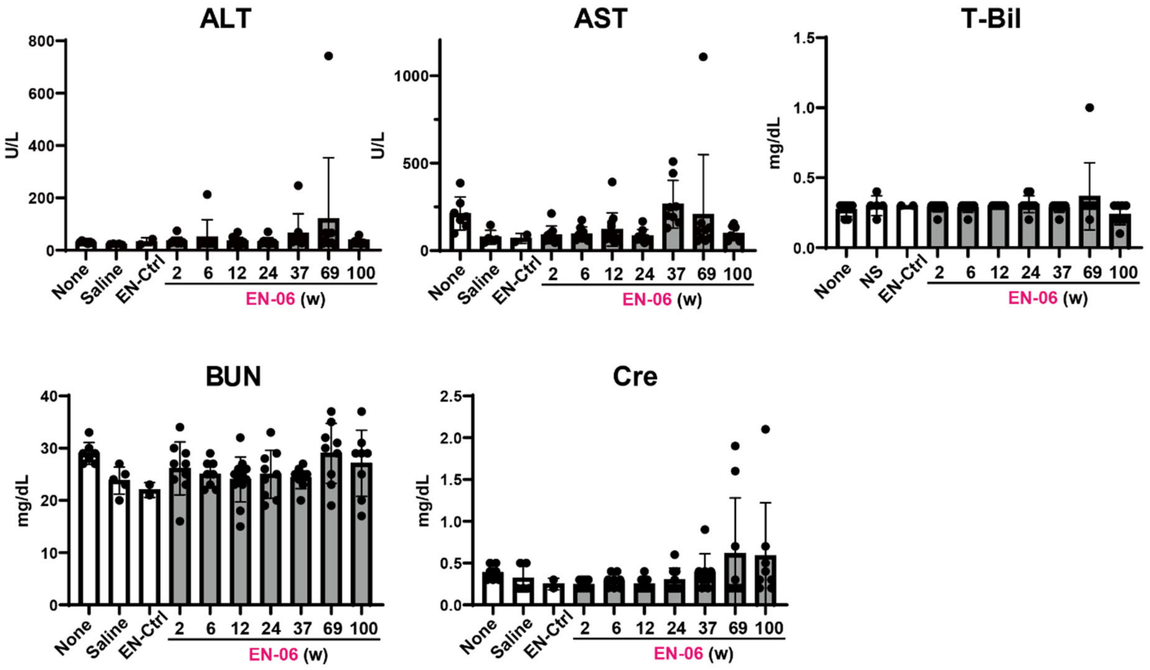

Figure S6. EN-06 has no significant negative effects on liver and renal function.

A panel of blood biochemical analytes associated with liver and renal function was assessed at various time points (2, 6, 12, 24, 37, 69, and 100 weeks) post-EN-06 administration. Mice administered the EN-Ctrl were assessed at 6 weeks, those administered the saline control at 2 weeks, and the non-injected control group was assessed immediately. Analytes measured included alanine aminotransferase (ALT), aspartate transaminase (AST), total bilirubin (T-Bil), blood urea nitrogen (BUN), and creatinine (Cre). There were no significant differences between the control group and any of the experimental groups (one-way ANOVA). The data shown represent the mean ± SD.

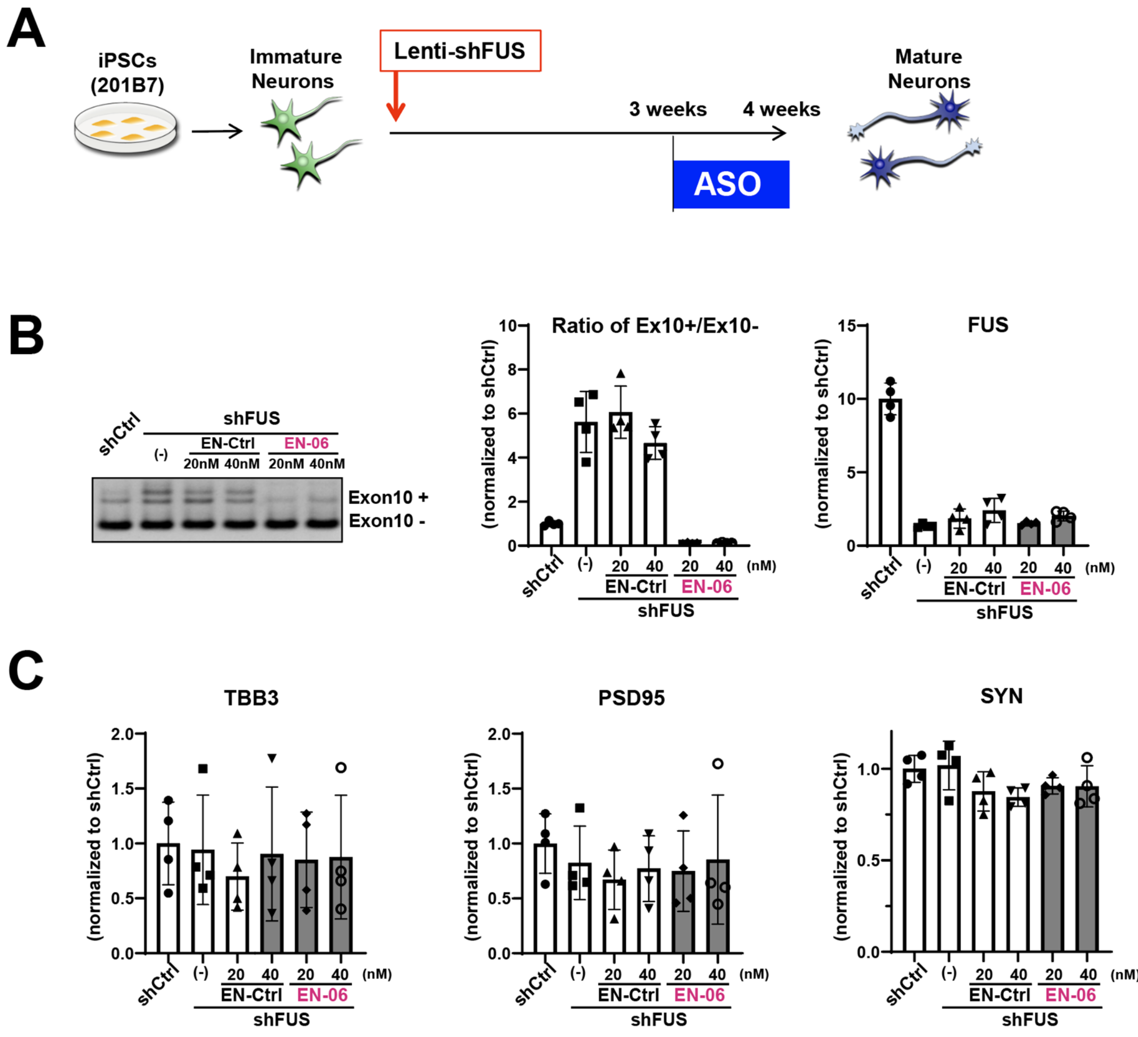

Figure S7. EN-06 restores the FUS-silencing induced increase in 4R-tau/3R-tau ratios in human-induced pluripotent stem cell (iPSC)-derived neurons. (A) Experimental scheme. Briefly, neurons infected with a lentivirus encoding shRNA were treated with 20 or 40 nM of each of the ASOs at 7 days post-differentiation. (B) Lentivirus-mediated shRNA targeting human FUS and a scramble control shRNA (shCtrl) were introduced into human iPSC-derived neurons. Neurons were then treated with 20 or 40 nM EN-06 or EN-Ctrl.

Alternative splicing of *MAPT* exon 10 was assessed by RT-PCR (left panel). The signals were quantified, and the isoform ratio was determined (middle graph). *FUS* expression was assessed by qRT-PCR (right graph). (C) Neuronal transcript levels of *βIII-tubulin* (TBB3), *PSD95*, and *synaptophysin* (SYN) were determined by qRT-PCR. The relative amount for each transcript was normalized to the housekeeping β-actin. The data shown represent the mean ± SD (n = 4 for each).

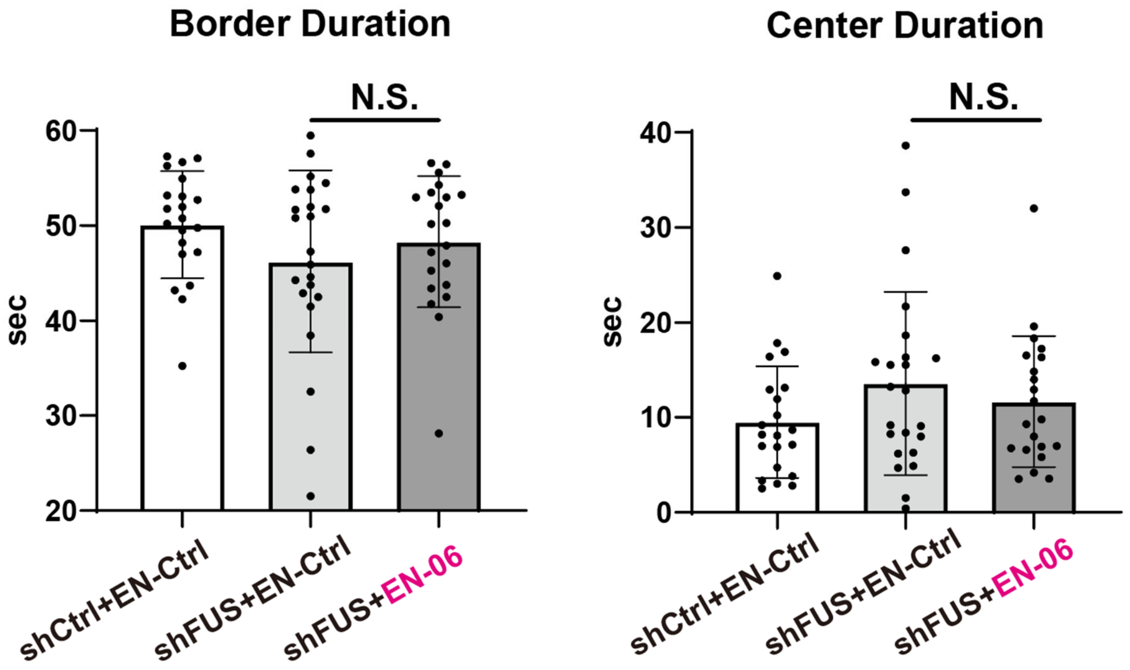

Figure S8. Open-field test behavioral assessment of the FUS knockdown hTau mouse model administered EN-06. Mice were treated with shCtrl + EN-Ctrl, shFUS + EN-Ctrl, or shFUS + EN-06. Time spent in the border (left graphs) and the center (middle graphs) area of an open field was determined (n = 21 for shCtrl + EN-Ctrl, n = 23 for shFUS +EN-Ctrl, and n = 21 for shFUS + EN-06; one-way ANOVA). N.S. denotes not significant. The data shown represent the mean ± SD.

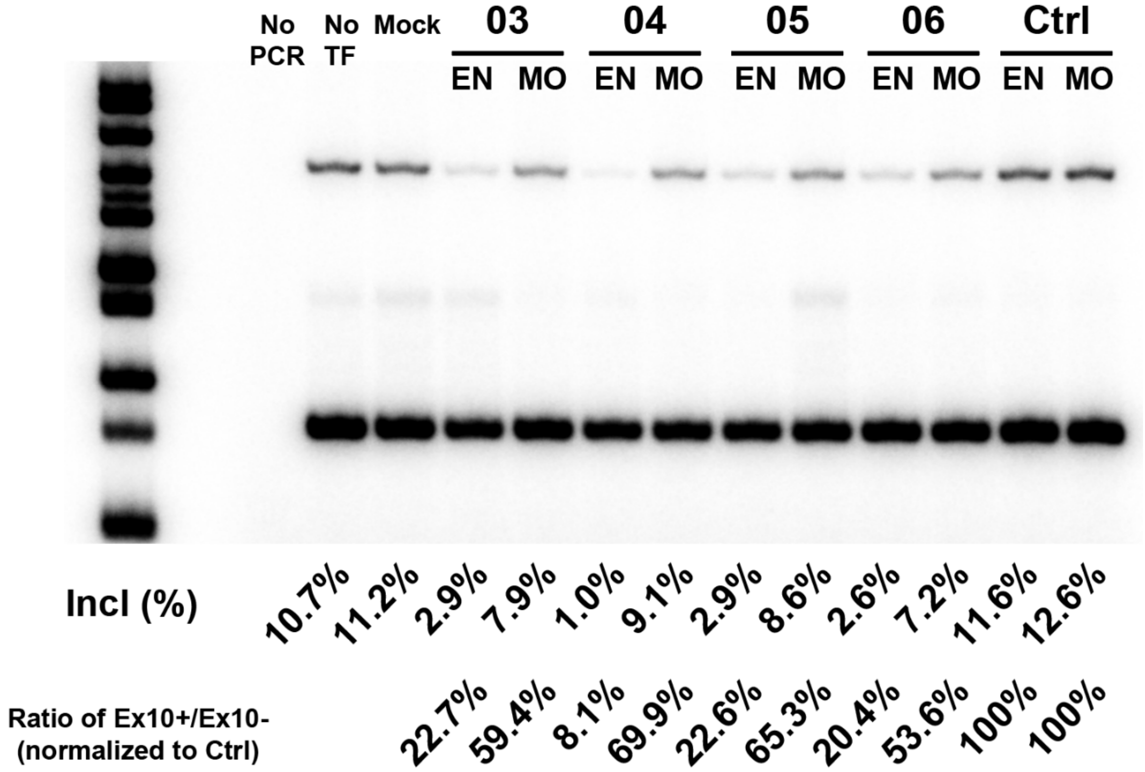

Figure S9. Efficiency of ENA- and MOE-modified ASOs targeting the same sequence to induce skipping of *MAPT* exon 10. RT-PCR was utilized to assess the effects of ENA- and MOE-modified ASOs (EN-03, MO-03, EN-04, MO-04, EN-05, MO-05, EN-06, and MO-06) and control ASOs (EN-Ctrl and MO-Ctrl) on the splicing of *MAPT* exon 10 in HEK293 cells. The percentage of exon 10-skipped transcripts was calculated relative to the total transcripts (sum of exon 10-skipped and exon 10-included transcripts) for each ASO. The ratio of exon 10-included to exon 10-skipped transcripts is shown, normalized to the ratio observed in the control, which was set to 100%.

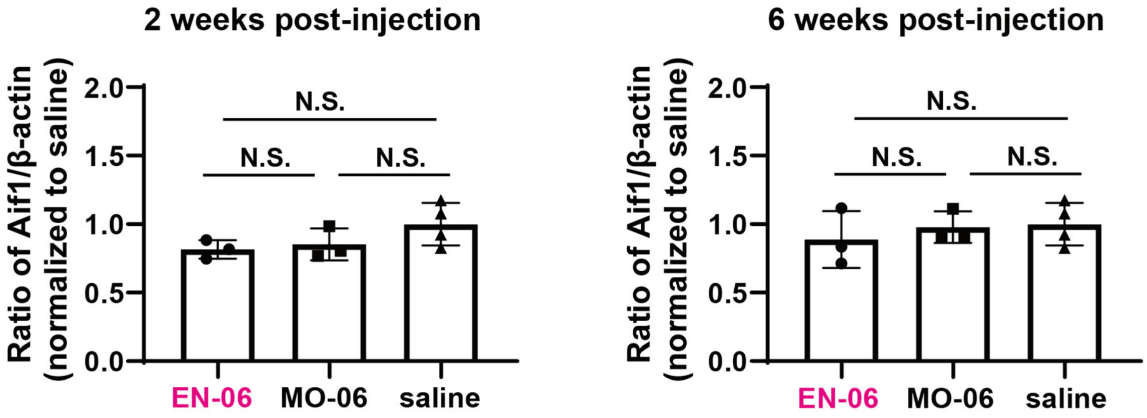

Figure S10. *Aif1* expression in the brain after ICV administration of ENA- or MOE-modified ASO-06.

The expression level of *Aif1*, which encodes the Iba1 protein, was assessed in brain homogenates of mice administered either EN-06 or MO-06. Data are shown as *Aif1* per *β-actin*; n = 3 for EN-06 and MO-06 and n = 4 for the saline control; one-way ANOVA). N.S. denotes not significant. The data shown represent the mean ± SD.

Table S1. ASOs used in this study.

Label	Sequence	Target	Function
EN-01	agCCagaaaaaaggaTga	Intron 9	Exon 10 skipping
EN-02	TggaCgTTgCTaagaTCC	Exon 10	Exon 10 skipping
EN-03	gCCaCaCTTggaCTggaC	Exon 10	Exon 10 skipping
EN-04	CCTTTgagCCaCaCTTgg	Exon 10	Exon 10 skipping
EN-05	TTaTCCTTTgagCCaCaC	Exon 10	Exon 10 skipping
EN-06	TaTTaTCCTTTgagCCaC	Exon 10	Exon 10 skipping
EN-07	gaTaTTaTCCTTTgagCC	Exon 10	Exon 10 skipping
EN-08	ggaCgTgTTTgaTaTTaT	Exon 10	Exon 10 skipping
EN-09	gCaTgggaCgTgTgaagg	Intron 10	Exon 10 inclusion
EN-10	gCaCggCgCaTgggaCgT	Intron 10	Exon 10 inclusion
EN-11	TTTaTTCTaTgCagTgTC	Intron 10	Exon 10 inclusion
EN-12	gCCCaagaaggaTTTaTT	Intron 10	Exon 10 inclusion
EN-13	ggCgCaTgggaCgTgTga	Intron 10	Exon 10 inclusion
EN-C1	CaTCTaagCaaCaaTTga	Non-specific	Control ASO
EN-C2 (EN-Ctrl)	CTCTTgaCgCaCaTCTgg	Non-specific	Control ASO
EN-C3	TTCCCTgaaggTTCCTCC	Non-specific	Control ASO
EN-C4	TCagTaaaCTTgaCaCCa	Non-specific	Control ASO

Uppercase letters: ENA-modified nucleotides

Lowercase letters: 2′-*O*-methylation-modified nucleotides

Table S2. Primers used for RT-PCR.

Gene	Forward	Reverse
mouse Fus	GGCTACTCCCAACAGAGCAG	GCTGTTTTGGGTCTGTCCAT
mouse β-actin	GCAAGTGCTTCTAGGCGGAC	AAGAAAGGGTGTAAAACGCAGC
mouse Aif1	AAGAGAGGCTGGAGGGGATC	GCTTCAAGTTTGGACGGCAG
human MAPT (exon10)	CCATGCCAGACCTGAAGAAT	TGCTCAGGTCAACTGGTTTG
human MAPT (whole)	GCAACATCCATCATAAACCAGGA	AGGGACCCAATCTTCGACTG
human FUS	AGCTCCCAATCGTCTTACGG	TTGCTGCTGTCCACCATAGC
human β-actin	GATCAAGATCATTGCTCCTCCT	GGGTGTAACGCAACTAAGTCA
human βIII-Tubulin	CATTCTGGTGGACCTGGAAC	ATACTCCTCACGCACCTTGC
human PSD95	ATATGTGAACGGGACCGAGG	TCACCGATGTGTGGGTTGTC
human SYN	GCCAACAAGACCGAGAGTGA	GAATTCGGCTGACGAGGAGT

Table S3. shRNAs used in this study.

shRNA Name	Target Sequence
FUS	GCAACAAAGCTACGGACAA
Ctrl	AATTCTCCGAACGTGTCACGT
