# Supplementary material for: Correcting tau isoform ratios with a long-acting antisense oligonucleotide alleviates 4R-tauopathy phenotypes
Source: Mol Ther Nucleic Acids. 2025 Mar 5;36(2):102503. doi: 10.1016/j.omtn.2025.102503 (PMC11979468; doi:10.1016/j.omtn.2025.102503)
Supplement: Document S2. Article plus supplemental information [file mmc2.pdf]

Molecular Therapy
Nucleic Acids
Original Article

Correcting tau isoform ratios with a long-acting antisense oligonucleotide alleviates 4R-tauopathy phenotypes

Kuniyuki Iwata-Endo,[1,2,11] Kentaro Sahashi,[2,11] Kaori Kawai,[2,3,11] Yusuke Fujioka,[1,2] Yohei Okada,[4] Eri Watanabe,[5] Nobuyuki Iwade,[2] Minaka Ishibashi,[2] Moniruzzaman Mohammad,[1] Asraa Faris Aldoghachi,[1] Dilina Tuerde,[2] Tsuyoshi Fujiwara,[6] Shinobu Hirai,[7] Haruo Okado,[7] Masahisa Katsuno,[2,3,8] Hirohisa Watanabe,[9] Kayoko Kanamitsu,[5] Masahiro Neya,[6] Shinsuke Ishigaki,[1,2] and Gen Sobue[10]

[1]Molecular Neuroscience Research Center, Shiga University of Medical Science, Otsu, Shiga 520-2192, Japan; [2]Department of Neurology, Nagoya University Graduate School of Medicine, Nagoya, Aichi 466-8550, Japan; [3]Institute for Glyco-core Research (iGCORE), Nagoya University, Furo-cho, Chikusa-ku, Nagoya 464-8601, Japan; [4]Department of Neural iPSC Research, Institute for Medical Science of Aging, Aichi Medical University, Nagakute, Aichi 480-1195, Japan; [5]Drug Discovery Initiative, Graduate School of Pharmaceutical Sciences, The University of Tokyo, Bunkyo-ku, Tokyo 113-0033, Japan; [6]KNC Laboratories Co., Ltd., Kobe, Hyogo 651-2271, Japan; [7]Department of Psychiatry and Behavioral Sciences, Tokyo Metropolitan Institute of Medical Science, Setagaya-ku, Tokyo 156-8506, Japan; [8]Department of Clinical Research Education, Nagoya University Graduate School of Medicine, Nagoya, Aichi 466-8550, Japan; [9]Department of Neurology, Fujita Health University, Toyoake, Aichi 470-1192, Japan; [10]Aichi Medical University, Nagakute, Aichi 480-1195, Japan

Tau, a microtubule-binding protein linked to tauopathies like Alzheimer's disease and frontotemporal lobar degeneration (FTLD), has 3-repeat (3R) and 4-repeat (4R) isoforms. Accumulation of the 4R-tau is associated with FTLD, progressive supranuclear palsy (PSP), and cortico-basal degeneration (CBD). We previously showed that a loss of fused in sarcoma (FUS) or splicing factor, proline- and glutamine-rich (SFPQ) promoted 4R-tau accumulation, which induced FTLD-like behaviors and neurodegeneration in mice. Here, we developed antisense oligonucleotides (ASOs) modified with 2′-*O*, 4′-*C*-ethylene-bridged nucleic acids (ENAs), reducing the 4R-tau/3R-tau ratio while maintaining total tau expression from the *MAPT* gene. *In vitro* screening identified EN-06 as the most effective ENA. Intracerebroventricular (ICV) administration of EN-06 corrected the 4R/3R-tau ratio in FUS-silenced humanized tau mice and human iPSC-derived neurons. This treatment ameliorated disease phenotypes, including aberrant behaviors, spine dysmorphology, and neurodegeneration. The half-life of EN-06 after a single ICV administration was approximately 6 months in the brain, with splicing correction effects that persisted for 2 years. The efficacy of EN-06 was higher than that of 2′-*O*-methoxyethyl (MOE)-modified ASO (MO-06). These findings highlight the potential of ENA-modified ASOs to reduce 4R-tau while preserving total *MAPT* expression, thus offering a safe and long-acting treatment for 4R-tau-associated tauopathies.

INTRODUCTION

Tau is a microtubule-binding protein that is causative for Alzheimer's disease (AD) and related tauopathies, including frontotemporal lobar degeneration (FTLD), progressive supranuclear palsy (PSP), and cortico-basal degeneration (CBD), all of which are characterized by an accumulation of phosphorylated tau. Among tauopathies, the 4-repeat (4R)-tau/3-repeat (3R)-tau ratio is markedly elevated in the brains of patients with FTLD, PSP, and CBD, collectively referred to as 4R tauopathies.[1–4] Patients with these intractable disorders are typically sporadic and manifest progressive cognitive dysfunction with or without symptoms of parkinsonism.[5] The therapeutic landscape for tauopathies encompasses immunotherapies and chemical compounds targeting tau expression, synthesis, phosphorylation, aggregation, and other related processes. Most are under preclinical or discovery stages, with a limited number in phase III trials.[6]

We previously reported that dysregulated alternative splicing of *MAPT* exon 10, which arises from the impaired formation of a fused in sarcoma (FUS) and splicing factor, proline- and glutamine-rich (SFPQ) protein complex, drives the increased 4R-tau/3R-tau ratio in FTLD.[7–9] A pathophysiological link between FUS and tau that impacts the regulation of the 4R-tau/3R-tau ratio is supported by familial FTLD cases characterized by astrocyte-predominant tauopathy, aberrant tau isoform ratio, and a Q140H substitution in the *FUS* gene.[10] Furthermore, the introduction of

Received 23 September 2024; accepted 28 February 2025;
https://doi.org/10.1016/j.omtn.2025.102503.

[11]These authors contributed equally

Correspondence: Shinsuke Ishigaki, Molecular Neuroscience Research Center, Shiga University of Medical Science, Otsu, Shiga 520-2192, Japan.
E-mail: shinsuke@belle.shiga-med.ac.jp
Correspondence: Gen Sobue, Aichi Medical University, Nagakute, Aichi 480-1195, Japan.
E-mail: sobueg@aichi-med-u.ac.jp

© 2025 The Author(s). Published by Elsevier Inc. on behalf of The American Society of Gene and Cell Therapy.
This is an open access article under the CC BY license (http://creativecommons.org/licenses/by/4.0/).

antisense oligonucleotides (ASOs) that elevate 4R-tau/3R-tau ratios causes abnormal nesting behavior in mice, which mimics executive cognitive dysfunction in FTLD.[11] The stronger affinity of 4R-tau for microtubules, compared with 3R-tau has physiological consequences that impact neuronal functions including axonal transport and mouse behaviors.[11–15] Thus, an increase in 4R-tau may presumably affect neurite extension and maintenance by impairing microtubule mobility. Indeed, we demonstrated that an adeno-associated virus (AAV) expressing short hairpin RNA (shRNA) against 4R-tau normalized 4R-tau levels and resulted in the recovery of mice with FTLD-like phenotypes. This suggests that repairing the 4R-tau/3R-tau ratio imbalance can be therapeutic for 4R tauopathies.[7]

ASOs designed to base-pair with a given pre-mRNA or mRNA sequence can impact the regulation of alternative splicing and gene silencing and have been employed as disease-modifying agents. A 2′-*O*-methoxyethyl (MOE)-sugar-modified ASO, nusinersen, is a disease-modifying ASO drug for spinal muscular atrophy (SMA) that restores alternative splicing of *SMN2* exon 7 by masking the hnRNP A1/A2 binding sites located in *SMN2* intron 7.[16–19] Its favorable pharmacokinetic/pharmacodynamic (PK/PD) properties and acceptable safety profiles significantly improved clinical outcomes in infants and children with SMA. The MOE moiety has also been used to promote the skipping of human *MAPT* exon 10 in mice expressing mutant tau transgenes[11]; however, the efficacy of the approach for reducing tauopathy phenotypes in animals has yet to be fully demonstrated.

To further enhance *in vivo* nuclease resistance (i.e., biostability), RNA binding affinity, and PK of ASOs, an extensive series of sugar modifications have been developed. One such modification, 2′-*O*,4′-*C*-ethylene-bridged nucleic acid (ENA), has been reported to exhibit both high nuclease resistance and biostability.[20] To specifically regulate alternative splicing of *MAPT* exon 10, we developed a suite of ENA-modified ASOs to target human *MAPT* exon 10 while minimizing effects on total tau expression levels and the negative impacts on neuronal function that arise with complete depletion of tau.[21,22] A screen of ENA-modified ASOs identified a suitable candidate ASO, termed EN-06, that efficiently induced the skipping of *MAPT* exon 10 and decreased the 4R-tau/3R-tau ratio. Intracerebroventricular (ICV) administration of EN-06 exerted beneficial effects on FTLD-like phenotypes and histopathological features observed in humanized tau mice in which the 4R-tau isoform was elevated following FUS silencing. Furthermore, compared with MOE-modified ASO with the identical base composition, the ENA-modified ASO exerted potent and durable skipping of *MAPT* exon 10 in the mouse brain with limited hepatic or renal toxicity, a potential adverse effect associated with ASO chemistries. As demonstrated here, the advantageous properties of ENA-modified ASOs hold significant potential for clinical therapeutic applications in familial and sporadic cases of tau-associated diseases.

RESULTS

Sequence determination of ENA-modified antisense oligonucleotides that regulate the splicing of *MAPT* exon 10

By taking advantage of the high-affinity binding of ENAs, we initially designed ENA-modified ASOs to hybridize to regions of human *MAPT* exon 10 and the flanking introns predicted to regulate alternative splicing of exon 10.[23] Ten ASOs that induced exon 10 skipping and three that promoted exon 10 inclusion were selected as candidates for further analysis along with four scrambled ASO controls (Figure 1A; Table S1). Each ASO was introduced into HEK293 cells and their effect on alternative splicing of *MAPT* exon 10 was analyzed by semiquantitative radiolabeled RT-PCR. Compared with controls, six ASOs (EN-02∼07) decreased the ratio of transcripts containing exon 10 by more than 80% as compared with the non-transfected control. In contrast, two ASOs (EN-11 and EN-12) increased the exon 10 inclusion ratio by more than 50% (Figure 1B).

Based on the antisense potencies and diversity of targeted sequences, three ENA-modified ASOs (EN-02, 03, and 06) that induced skipping of *MAPT* exon 10 *in vitro* were arbitrarily chosen for *in vivo* screening. EN-C2 was selected as the control ASO (EN-Ctrl). To investigate their skipping effects, we performed ICV injections using each of the ASOs or a control ASO in 6-week-old hTau mice (TAU KO; hT-PAC-N), which produce the normal human tau gene (*MAPT*) without endogenous tau production. RT-PCR revealed that EN-02, 03, and 06 significantly decreased the ratio of transcripts with exon 10 included in the brain at 6 weeks post-injection by 55%, 92%, and 74% as compared with the control ASO, respectively. The ASOs also reduced the total amount of *MAPT* transcripts by 27% (EN-02), 47% (EN-03), and 14% (EN-06) (Figure 1C). EN-06, which reduced 4R-tau, while largely maintaining total tau expression, was selected for *in vivo* investigation (Figure S1).

A single ICV injection of EN-06 efficiently skipped *MAPT* exon10 in the mouse brain

To optimize the efficacy of the ICV injection, we next examined the dose-response of EN-06. At 6 weeks post-injection, RT-PCR showed that skipping of *MAPT* exon 10 in the hippocampus increased as the EN-06 dose increased from 0 to 100 μg, with the skipping potency most pronounced at 50 μg. A similar trend was observed by the immunoblotting (Figure 2A). At 200 μg, EN-06 also reduced total *MAPT* mRNA expression, indicating that an excess amount of EN-06 arrests *MAPT* transcription. We observed only one case of accidental death following ASO injections (one death out of 67 mice treated with EN-06), indicating that compound-specific acute toxicity was not evident. Since neuroinflammation can be provoked by ASO chemistry,[24,25] we measured the expression levels of *Aif1*, a marker of monocyte and microglia activation, in the brains of mice administered various EN-06 doses. Apart from 200 μg EN-06, ICV administration did not increase *Aif1* mRNA levels (Figure S2). Based on these results, we selected 50 μg EN-06 as the optimal dose for a single ICV injection in adult hTau mice.

To analyze the *in vivo* duration of ASO action, we assessed the impact of EN-06 on *MAPT* exon 10 skipping at various time points post-injection.

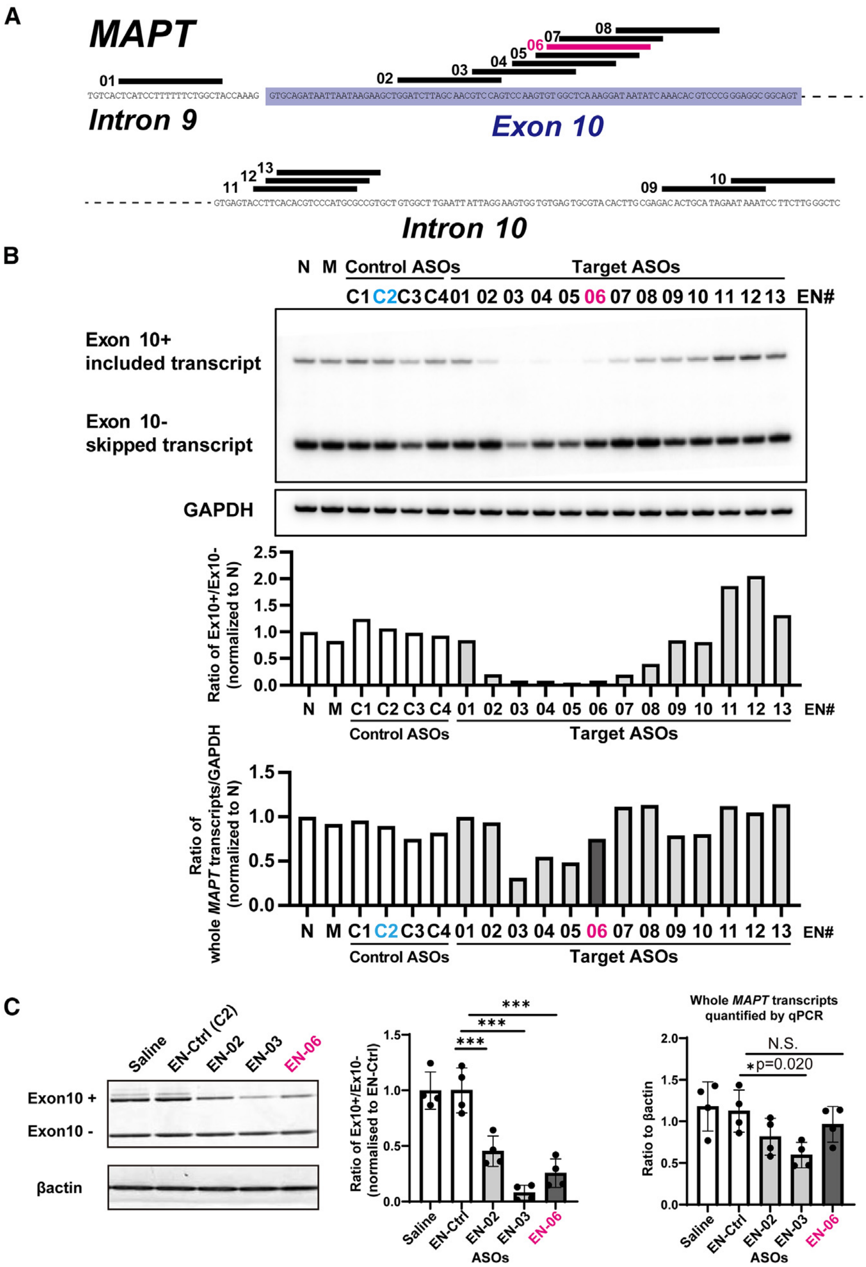

Figure 1. Design and screening of ENA-ASO candidates that target human *MAPT* exon 10

(A) Schematic diagram of human *MAPT* exon 10 and the flanking intronic regions with the ENA-ASO annealing sites indicated by lines over the complementary sequence. EN-06 is shown in magenta. (B) RT-PCR screening of ASO-mediated *MAPT* exon 10 skipping. Upper panel: representative gel image. Middle panel: the ratio of exon 10-included transcripts to exon 10-skipped transcripts. Lower panel: the ratio of total *MAPT* gene transcripts (sum of exon 10-included transcripts and exon 10-skipped transcripts) to GAPDH. These data illustrate the effects of the 17 ASOs (13 target ASOs and four control ASOs) on the splicing of *MAPT* exon 10 in HEK293 cells. N: no transfection; M: mock transfection; Ex10+: exon 10-included transcripts; Ex10-: exon 10-skipped transcripts. (C) The effects of EN-02, EN-03, and EN-06 on *MAPT* exon 10 skipping at 4 to 6 weeks post-injection in the hippocampus. Single ICV injections of each ASO, control ASO (EN-Ctrl, equivalent to EN-C2), or saline were made into 6-week-old hTau mice (TAU KO; hT-PAC-N). Left panel: representative image of hippocampal RT-PCR showing *MAPT* exon 10-included and skipped bands and bands of *β-actin* as a loading control. Middle panel: the ratio of exon 10-included transcripts to exon 10-skipped transcripts. Right panel: total *MAPT* transcript levels measured by qPCR and normalized to *β-actin* levels. For each group $n = 4$, statistical differences were assessed via one-way ANOVA (*$p < 0.05$, ***$p < 0.001$). N.S. denotes not significant. Data are mean ± SD. Ex10+: exon 10-included transcripts; Ex10−: exon 10-skipped transcripts.

EN-06 (50 μg) was injected via ICV into 6-week-old hTau mice, and the ratio of 4R-tau to 3R-tau isoforms in the hippocampus was examined at 2, 6, 12, and 24 weeks post-injection. Both RT-PCR and immunoblotting showed that exon 10 skipping persisted for up to 24 weeks (Figure 2B).

Pharmacokinetics/pharmacodynamics of ICV-administered EN-06

To investigate the tissue distribution of EN-06, mice were administered 50 μg of FITC-labeled EN-06 via ICV. The EN-06 persisted for 10 weeks, and while distributed throughout the brain, it was most concentrated in the hippocampus (Figure S3A). To validate this finding, we developed a specific antibody against EN-06 (Figure S3B). Immunofluorescent staining confirmed that EN-06 could be specifically and dose-dependently detected (Figure S3C), and indeed showed that EN-06 distributed throughout the brain. Localization, again, was most notable within hippocampal neurons at 6 weeks post-injection following administration of a single 50-μg dose (Figure 3A).

To quantify the absolute tissue concentration of EN-06 in the hippocampus, we developed a method combining solid-phase extraction with liquid chromatography-mass spectrometry (LC-MS) analyses. At 6 weeks post-injection of a single 50-μg dose, EN-06 was stably isolated at approximately 500–700 pMol/g of brain tissue (Figure S4A). To assess the correlation between the LC-MS quantification and the immunofluorescent data, we measured the signal intensities of EN-06 in the hippocampus sections at 6 weeks post-injection. The EN-06 signals in the hippocampus increased as the amount injected increased, and the signal intensities were comparable to the LC-MS quantification data, with a significant correlation between

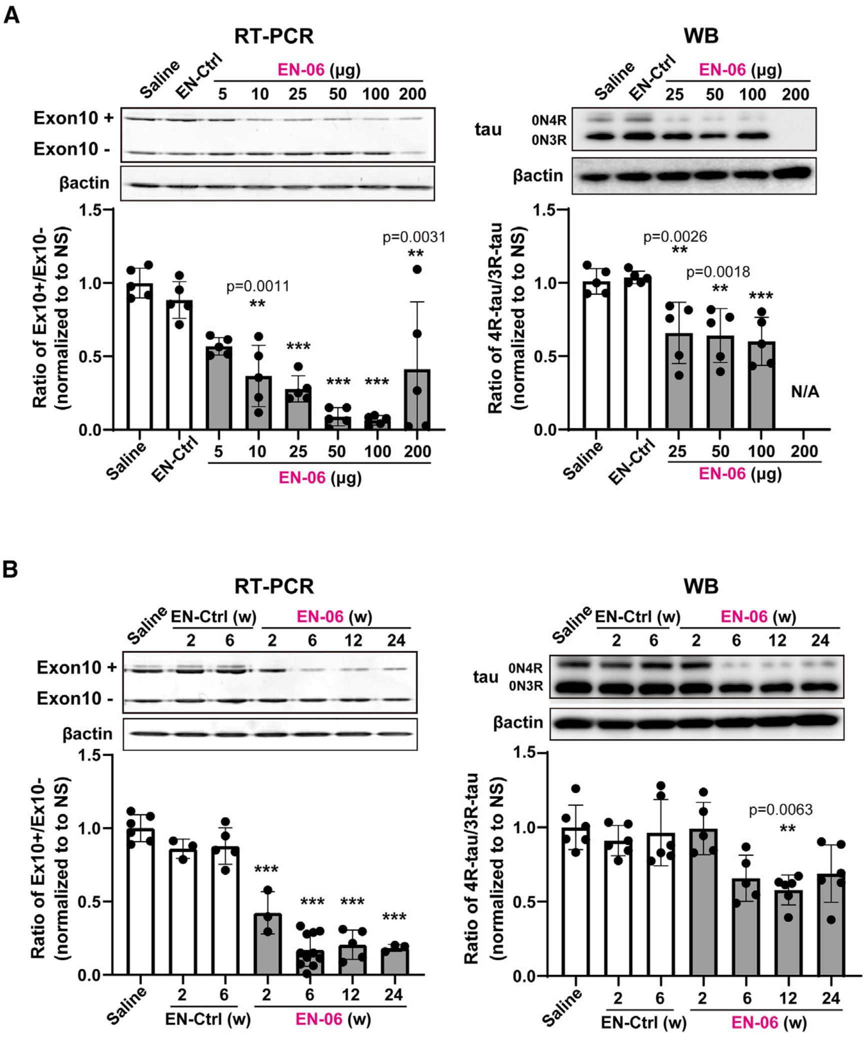

Figure 2. Dose dependence and persistence of EN-06 in humanized tau mice following a single ICV administration

(A) Dose-response of EN-06 following a single ICV injection. RT-PCR analysis effects of *MAPT* exon 10 skipping in the hippocampus were assessed at 6 weeks post-injection (representative images shown in the upper left panel). Quantification of the RT-PCR band intensities (bottom left graph, n = 5 for each; one-way ANOVA). Immunoblot analysis of the same samples (representative images shown in the upper right panel). Quantification of the immunoblot band intensities (bottom right graph, n = 5 for each; one-way ANOVA). (B) Persistence of EN-06 effects of *MAPT* exon 10 skipping over time in the hippocampus. ICV injection of 6-week-old hTau mice with 50 μg EN-06. The expression levels of 4R-tau and 3R-tau in the hippocampus were analyzed by RT-PCR and immunoblot at 2, 6, 12, and 24 weeks post-injection. Representative RT-PCR gel images are shown in the upper left panel with quantification data shown in the lower left graph. Saline injection for 2 weeks (n = 6); EN-Ctrl - control ASO for 2 weeks (n = 3) and 6 weeks (n = 5); EN-06 for 2 weeks (n = 3), 6 weeks (n = 11), 12 weeks (n = 5), and 24 weeks (n = 3). Representative immunoblot images are shown in the upper right panel with quantification data shown in the lower right graph. Saline injection for 2 weeks (n = 6); EN-Ctrl - control ASO for 2 weeks (n = 6) and 6 weeks (n = 6); EN-06 for 2 weeks (n = 5), 6 weeks (n = 5), 12 weeks (n = 6), and 24 weeks (n = 6). β-actin bands are shown as a loading control. Statistical differences were assessed via one-way ANOVA (**$p < 0.01$, ***$p < 0.001$). Data are mean ± SD. Ex10+: exon 10-included transcripts; Ex10−: exon 10-skipped transcripts.

the two methods (Figures S4B–S4D). Following a single 50-μg ICV injection, the concentration of EN-06 in the cortex and hippocampus exceeded 200 pMol/g brain for up to 12 weeks post-injection. The concentration in the liver was also high at 2 and 6 weeks post-injection (644 and 530 pMol/g liver, respectively) but dropped below 200 pMol/g liver after 6 weeks post-injection. The half-lives of EN-06 in the cortex and hippocampus were 22.1 and 25.7 weeks, respectively, whereas those in the liver and kidney were 4.7 and 8.4 weeks, respectively (Figure 3B).

Immunohistochemistry similarly revealed that EN-06 signals in the hippocampus diminished over time (Figure S4E).

ICV-administered ASOs can be localized in the liver and kidneys for a few weeks post-injection.[26] We thus examined the peripheral organ tissue distribution of EN-06. While EN-06 was evident in the liver at 2 weeks post-injection, it was barely detectable in the kidney, cardiac muscles, and spleen (Figure S5A). EN-06 signals in hepatic cells, however, markedly decreased at 6 weeks post-injection and were undetectable at 12 weeks post-injection (Figure S5B).

Based on the persistence of the signals above and the sustained skipping of *MAPT* exon 10 (Figure 2B), we next examined the prolonged effects of EN-06 at 1 and 2 years post-injection. Notably, the skipping effects of EN-06 were evident in the brains of mice at both time points (Figure 3C).

We also evaluated the impact of EN-06 on non-neuronal organs by monitoring serum markers in mice over time. The levels of the liver function markers aspartate aminotransferase (AST), alanine aminotransferase (ALT), and total bilirubin in EN-06 mice were comparable to the saline controls (Figure S6). Similar results were seen with the kidney function markers creatinine and blood urea nitrogen (Figure S6). Taken together, these data indicate that EN-06 does not induce hepatic or renal damage.

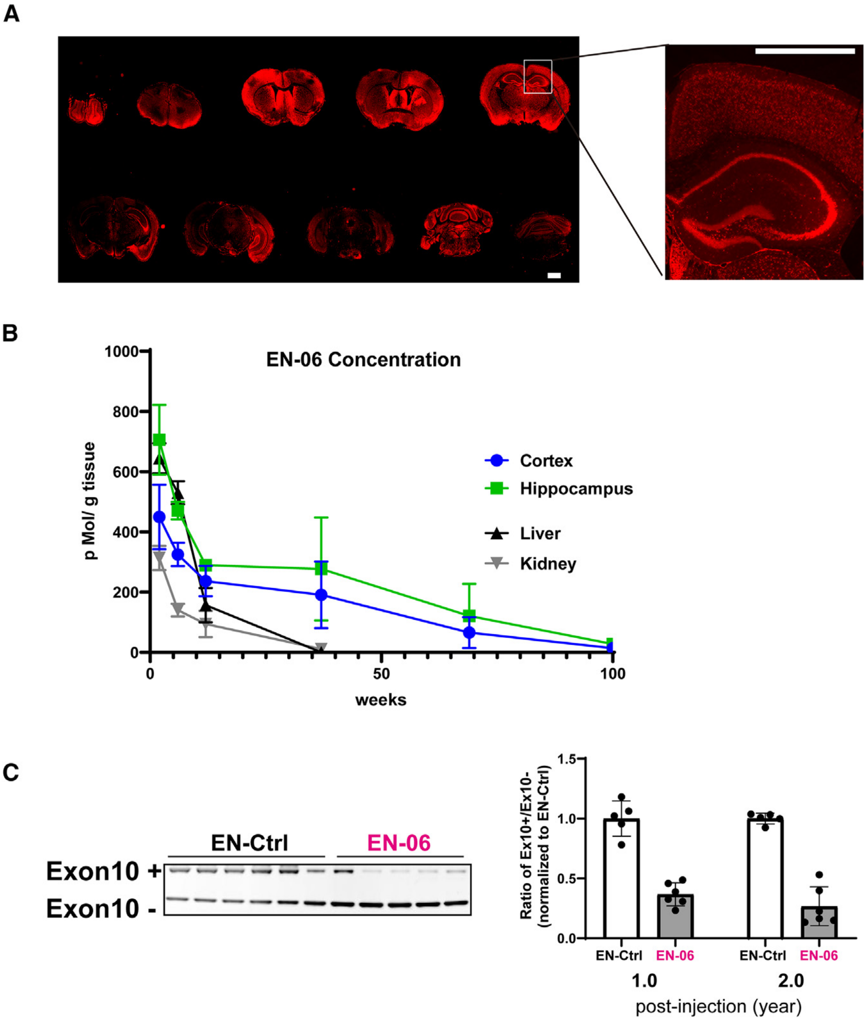

Figure 3. Pharmacokinetics/pharmacodynamics of ICV-administered EN-06

(A) Immunofluorescent imaging of EN-06 distribution in the brain. Mouse brain sections were stained using a custom antibody against EN-06 at 6 weeks post-ICV injection of 50 μg EN-06 (left). The inset depicts a high-magnification image. Scale bars, 1.0 mm. (B) Time-dependent concentration of EN-06 in various tissues. EN-06 levels determined via LC-MS after a single 50-μg ICV injection of EN-06. Tissues analyzed include the following: cortex at 2 (n = 4), 6 (n = 4), 12 (n = 5), 37 (n = 8), 69 (n = 6), and 100 weeks (n = 6); hippocampus at 2 (n = 4), 6 (n = 4), 12 (n = 5), 37 (n = 8), 69 (n = 6), and 100 weeks (n = 6); liver at 2 (n = 3), 6 (n = 3), 12 (n = 3), and 37 weeks (n = 3); kidney at 2 (n = 3), 6 (n = 3), 12 (n = 3), and 37 weeks (n = 4). No EN-06 was detected (<0.6 nMol/L) in serum at 2 weeks post-injection and all subsequent time points. Data are mean ± SEM. (C) EN-06 is stable in brain tissue and its effects persist for up to 2 years post-injection. The long-term effects of EN-06 were evaluated using RT-PCR at 1 and 2 years post-injection. A representative RT-PCR gel image at 2 years post-injection is shown in the left panel and the quantified ratios of exon 10-included transcripts (Ex10+) to exon 10-skipped transcripts (Ex10−) are shown in the right panel. EN-Ctrl at 1 year (n = 5), EN-06 at 1 year (n = 6), EN-Ctrl at 2 years (n = 6), and EN-06 at 2 years (n = 5). Statistical differences were assessed with Welch's t test (***p < 0.001). Data are mean ± SD.

Effects of EN-06 on *MAPT* exon 10 splicing in FUS-silenced human iPSC-derived neurons

To test the skipping potency of EN-06 in a human FTLD neuronal model, we reduced the expression of FUS in human-induced pluripotent stem cell (iPSC)-derived neurons using a lentivirus expressing shRNA against endogenous FUS and then treated with EN-06 for 7 days (Figure S7A). As expected, FUS silencing induced an increase in the 4R-tau/3R-tau ratio, and EN-06 treatment restored the ratio (Figure S7B). EN-06 treatment had no effect on the expression of genes associated with neuronal differentiation (Figure S7C).

A single ICV injection of EN-06 ameliorated aberrant FTLD-like behaviors in FUS-silenced hTau mice

MAPT undergoes FUS-dependent altered splicing both *in vitro* and *in vivo*.[7,15] As a model for FTLD, we generated hippocampal silenced FUS in an hTau mouse background by stereotaxic injection of an AAV expressing shRNA against endogenous murine FUS (AAV-shFUS) into the bilateral hippocampus of 6-week-old mice. Control hTau mice were generated by injecting a control AAV (AAV-shCtrl). Both RT-PCR and immunoblotting showed that FUS silencing increased the 4R-tau/3R-tau ratio (Figures 4A and 4B).[7] A single ICV injection of 50 μg EN-06 in 6-week-old shFUS mice efficiently induced the skipping of *MAPT* exon 10 and normalized the 4R-tau/3R-tau ratio without causing a significant change in total tau levels (Figures 4A and 4B).

To investigate the beneficial effects of EN-06 on behavioral impairments caused by FUS silencing, mice were subjected to behavioral analysis at 6 weeks post-injection. As reported previously,[7,27] shFUS mice are characterized by a significant increase in the entry on open arms in the elevated-plus maze (EPM) test, which is consistent with the anxiety observed in FTLD patients. A single ICV injection of EN-06 rescued these impaired behaviors (Figure 4C). An open field test showed that EN-06 treatment tended to decrease the duration spent in the center area, which potentially reflects suppression of the abnormal behavioral disinhibition observed in patients (Figure S8).

We recently found that feeding behavior patterns could be an early biomarker for psychosocial stress and neuropsychiatric disease conditions in mice.[28] Aberrant feeding behavior patterns have been

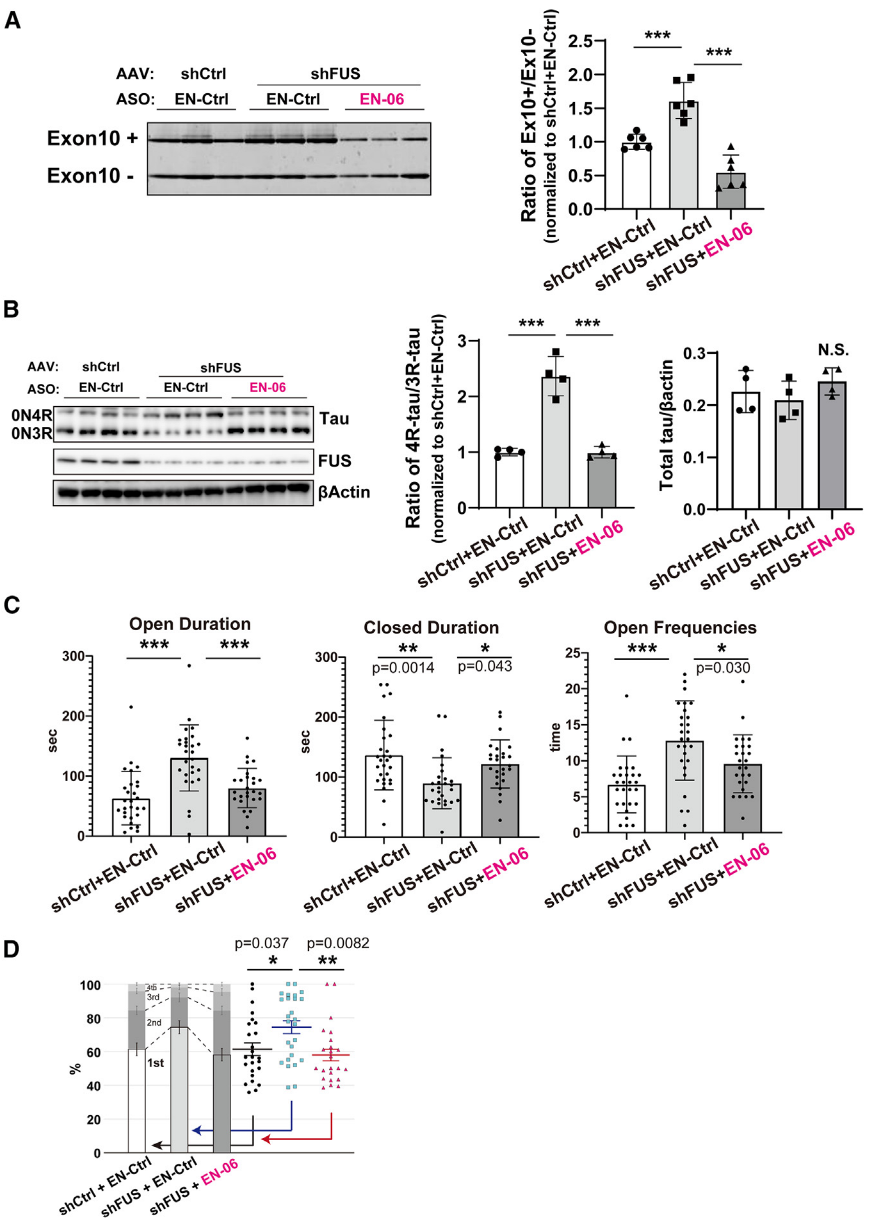

Figure 4. EN-06 ameliorates FTLD-like phenotypes in mice by reducing the 4R-tau/3R-tau ratios induced by FUS silencing

(A) Hippocampal RT-PCR of mice treated with AAV expressing shCtrl + EN-Ctrl, shFUS + EN-Ctrl, or shFUS + EN-06 (left). The quantified ratios of the exon 10-included transcripts (Ex10+) to exon 10-skipped transcripts (Ex10−) were determined (right: n = 6 for each, one-way ANOVA). (B) Hippocampal immunoblotting of mice treated as in (A) (left) with the quantified 4R-tau/3R-tau ratios determined (middle: n = 4 for each, one-way ANOVA). The levels of total tau proteins were quantified from the WB images and normalized to the corresponding actin levels (right: n = 4 for each, one-way ANOVA). (C) Elevated-plus maze test of mice treated as in (A). Time spent in the open (left graphs) and the closed (middle graphs) arm of an elevated-plus maze and the number of entries into the open arm (right graphs) were determined for each group (n = 28 for shCtrl + EN-Ctrl, n = 27 for shFUS + EN-Ctrl, and n = 26 for shFUS + EN-06; one-way ANOVA). (D) The spatial feeding patterns of mice treated as in (A). The duration at each bait location was determined based on the amount consumed and then each location was ranked in decreasing order in terms of bait consumption and a preference ratio was plotted. Individual measurements used to determine the preferred bait source ratios are shown to the right of the histogram. Statistical analysis was performed on the preference ratio (n = 25 for shCtrl + EN-Ctrl, n = 26 for shFUS + EN-Ctrl, and n = 23 for shFUS + EN-06; one-way ANOVA).

reported in FTLD patients.[29–31] Consistent with this, we found that shFUS mice exhibit fixated feeding, in which a mouse focuses on a specific bait location. Administration of EN-06 rescued this aberrant feeding behavior in the FUS-silenced mice (Figure 4D).

A single ICV injection of EN-06 rescued the early-stage synaptic abnormalities, late-stage neurodegeneration, and tau phosphorylation observed in shFUS mice

We previously reported that a loss of FUS in mice results in abnormal spine formation, which is associated with the onset of abnormal behaviors at the early disease stage.[27,32] To investigate the histological state of dendritic spines in shFUS mice treated with EN-06, we performed Golgi-Cox staining. EN-06 normalized the proportional reduction of mature dendritic spines in hippocampal CA1 dendrites of 3-month-old shFUS mice (Figure 5A).

Significant hippocampal atrophy accompanied by neuronal cell loss and an accumulation of phosphorylated tau was observed in mice 18 months post-FUS silencing.[7] Since exon skipping was observed in hTau mice 2 years post-ICV injection of EN-06 (Figure 3C), we hypothesized that the prolonged skipping effect of EN-06 would similarly have beneficial impacts on shFUS mouse brains at 1 and 2 years post-injection. To assess the effects of EN-06 on the neurodegeneration caused by FUS silencing at the late disease stage, mice around 100 weeks old were subjected to histological analysis. As reported in our previous study,[7,27] shFUS mice administered a control ASO exhibited significantly greater hippocampal atrophy and neuronal loss as compared with mice administered the control ASO. A single administration of EN-06, however, significantly ameliorated both the hippocampal atrophy (Figure 5B) and neuronal loss (Figure 5C) induced by FUS silencing.

Immunohistostaining with a PHF1 antibody that is specific for phosphorylated tau revealed the presence of inclusion-like phosphorylated

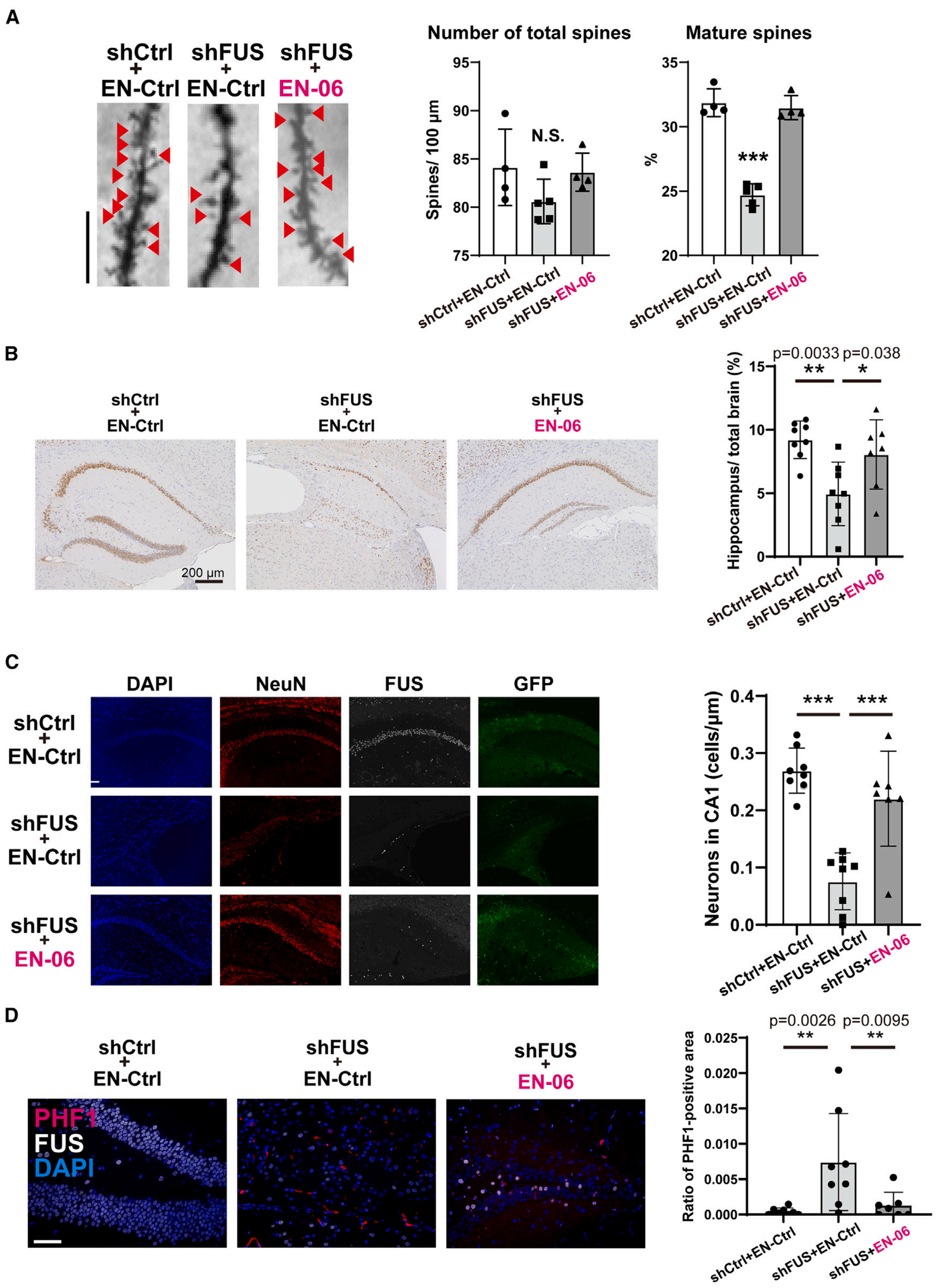

Figure 5. EN-06 restored disease phenotypes in both the early and late disease stages of shFUS mice

(A) Administration of EN-06 restored the abnormal dendritic spine morphology caused by FUS silencing that is characteristic of early-stage disease development. Representative images of CA1 pyramidal neuron dendritic segments isolated from the hippocampus of mice treated with AAV expressing shCtrl + EN-Ctrl, shFUS +EN-Ctrl, or

(legend continued on next page)

tau signals in the residual hippocampal neurons of shFUS mice administered the control ASO. The phosphorylated tau levels were elevated in shFUS mice relative to shCtrl mice but returned to control levels following EN-06 treatment (Figure 5D).

Comparison of the ENA-modified ASO and 2′-*O*-methoxyethyl (MOE)-ASO efficiencies on *MAPT* exon 10 splicing

To compare the efficiencies of different ASO chemistries on *MAPT* exon 10 skipping, ENA- or MOE-modified ASOs sharing the same bases were introduced into HEK293 cells. Exon skipping was enhanced by more than 2-fold with the ENA-modified ASOs as compared with the MOE-modified ASOs (Figure S9).

We next assessed the skipping potency in hTau mice by injecting via ICV a single 50-μg dose of EN-06 or an MOE-modified ASO with the same base sequence as EN-06 (MO-06) into mice at 6 weeks of age. RT-PCR revealed that EN-06 induced a 50% reduction in the 4R-tau/3R-tau ratio, which persisted for up to 100 weeks post-injection. In contrast, the reduction of the 4R-tau/3R-tau ratio in MO-06 injected mice was less pronounced, and the maximal effect only lasted 6 weeks before returning to control levels at 24 weeks post-injection (Figure 6A). Similar results were obtained when tau protein isoform levels were examined by immunoblotting (Figure 6B).

In addition to pharmacological efficacy, the *in vivo* safety and tolerability of ASOs must be evaluated before they can be considered for further clinical applications as antisense therapeutics. We used qPCR to examine the expression of the inflammation marker gene *Aif1* in the brain of mice injected via ICV with EN-06 or MO-06. *Aif1* mRNA levels in EN-06, MO-06, or saline-treated mice were comparable at 2 and 6 weeks post-injection, indicating that any chemistry-related neuroinflammation associated with the ENA or MOE moiety is limited (Figure S9).

DISCUSSION

While evaluating the efficacy of ENA-modified ASOs to induce skipping of *MAPT* exon 10 in the brain of an FTLD mouse model, we discovered that a single ICV injection of EN-06 achieved long-term normalization of the tau isoform ratio and alleviated FTLD-like symptoms.

Tauopathy is a pathological disease characterized by the deposition of phosphorylated tau and includes FTLD, PSP, CBD, and AD. A reduction in total tau levels by genetic engineering can ameliorate neuronal dysfunction and neuronal loss, and increase survival in mouse tauopathy models.[33,34] As new therapeutics are considered, the ability of ASOs to penetrate cells is particularly advantageous because current tau-targeting antibodies are limited to neutralizing extracellular protein, whereas tau is largely intracellular. Consequently, ASOs that target intracellular tau have been developed. Among the more promising ASOs is the MOE-ASO, BIIB080, which reduced both *MAPT* mRNA levels and tau pathology. It also prevented hippocampal volume loss and increased the survival of tau transgenic mice.[35] In contrast, antibody-based suppression of tau caused impaired motor function in AD mice.[36] Moreover, tau deletion leads to aberrant long-term depression and affects reward processing in mice.[21,22] Partial deletion of the *MAPT* gene was identified in a family with FTLD who exhibited compulsive consumption of cigarettes and alcohol.[37] Furthermore, tau depletion affects reward processing in mice,[22] and in *Drosophila* the loss of tau causes additive defects in behavioral responses to ethanol exposure.[38] These findings raise concerns that complete loss of tau could compromise neuronal function, leading to neuropsychiatric symptoms and cognitive deficits.

Compared with RNA degradation mediated by RNase H cleavage or RNA interference mechanisms, such as the approach with BIIB080, which impacts total tau levels, a strategy that modulates splicing would only act on selected isoforms while maintaining total target transcripts. This approach would thus preserve the physiological function of the target gene. We consequently developed an ASO strategy applicable for all familial and sporadic cases of PSP, CBD, and FTLD in which 4R-tau accumulates. This intervention would reduce 4R-tau levels by correcting *MAPT* exon 10 splicing without compromising tau expression in general. Initially identified through *in vitro* screening, the candidate ASO, EN-06, restored the altered 4R-tau/3R-tau ratio caused by FUS silencing in hTau mice and human iPSC-derived neurons. Without inducing acute and chronic toxicity, EN-06 treatment also reversed the physiological manifestations of early-stage disease in shFUS mice, including FTLD-like aberrant behavior patterns and abnormal morphology

shFUS + EN-06 mice (left images; 10 dendrites were assessed using four mice from each treatment group). Mushroom spines are indicated by arrowheads. The spine number was quantified along a 100-μm segment from the origin of the primary apical dendritic branches of Golgi-impregnated CA1 pyramidal neurons. Both total spine abundance (middle graph) and the ratio of mushroom-shaped, mature spines to total spines (right graph) were determined. (B) Representative histopathology images of mice treated with AAV expressing shCtrl + EN-Ctrl, shFUS + EN-Ctrl, or shFUS + EN-06 at 24 months post-injection. Sections were stained with an anti-NeuN antibody. Scale bar, 200 μm. Hippocampal volume was quantitatively determined using NeuN-stained coronal sections of mice treated with AAV expressing shCtrl + EN-Ctrl, shFUS + EN-Ctrl, or shFUS + EN-06 at 24 months post-injection (right graph, $n = 8$ for shCtrl + EN-Ctrl, $n = 8$ for shFUS + EN-Ctrl, and $n = 7$ for shFUS + EN-06; one-way ANOVA; $^*p < 0.05$, $^{**}p < 0.01$). (C) Representative immunofluorescent images of the CA1 sector from the hippocampus of mice treated with AAV expressing shCtrl + EN-Ctrl, shFUS + EN-Ctrl, or shFUS + EN-06 at 24 months post-injection. Sections were stained with anti-NeuN, anti-FUS, and anti-GFP antibodies. Scale bar, 50 μm (left images). For quantification of neuronal loss in the hippocampus, the number of NeuN-stained cells in the CA1 sector of the hippocampus was determined (right graph, $n = 8$ for shCtrl + EN-Ctrl, $n = 8$ for shFUS + EN-Ctrl, and $n = 7$ for shFUS + EN-06; one-way ANOVA; $^{***}p < 0.001$). Data are mean ± SD. (D) To assess the degree of tau phosphorylation in the hippocampus, immunofluorescent imaging was done with a PHF1 antibody using the sections shown in (C). Representative immunofluorescent images of the hippocampus from mice treated with AAV expressing shCtrl + EN-Ctrl, shFUS + EN-Ctrl, or shFUS + EN-06 at 24 months post-injection. Sections were stained with PHF1 and anti-FUS antibodies. Scale bar, 50 μm (left images). The PHF1 positive area in the hippocampus was quantified (right graph, $n = 8$ for shCtrl + EN-Ctrl, $n = 8$ for shFUS + EN-Ctrl, and $n = 7$ for shFUS + EN-06; $^*p < 0.05$, $^{**}p < 0.01$, $^{***}p < 0.001$). N.S. denotes not significant. Data are mean ± SD.

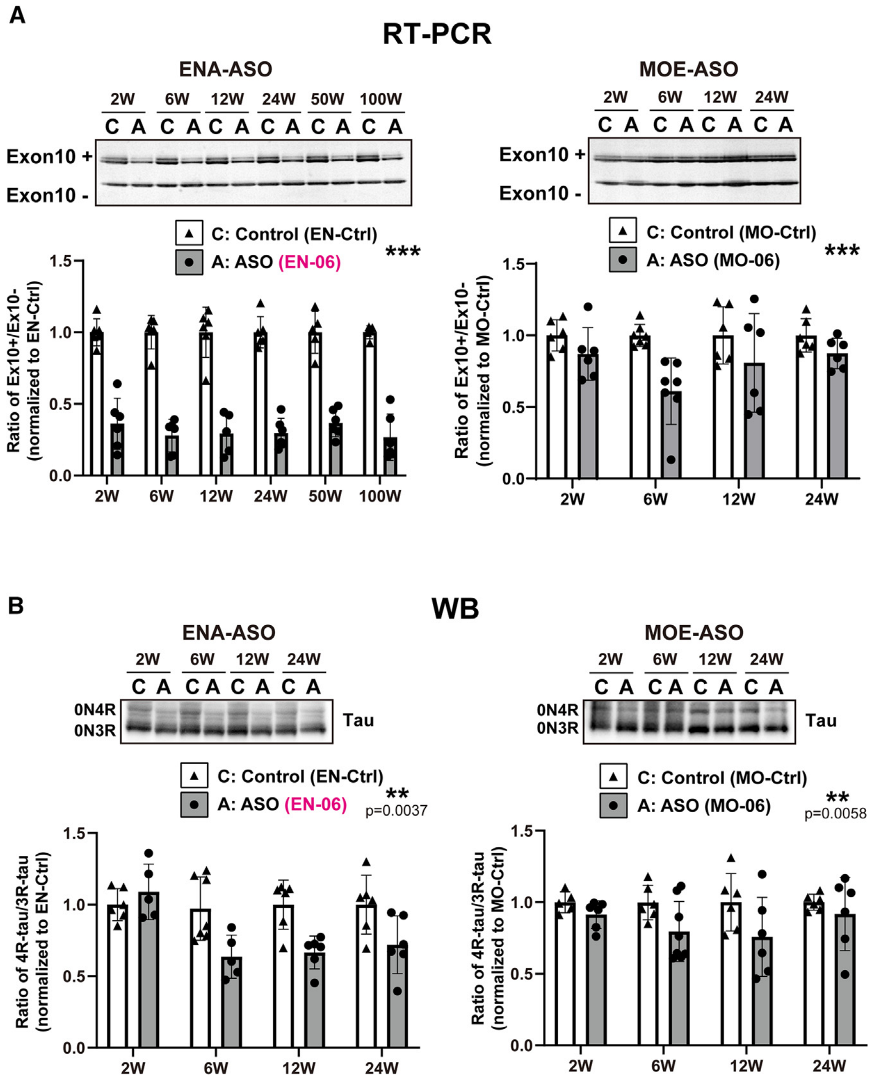

Figure 6. Comparison of potencies between ENA-ASO and MOE-ASO that target *MAPT* exon 10

(A) Time course comparison of EN-06 and MO-06 effects of *MAPT* exon 10 skipping. Six-week-old hTau mice were injected via ICV with 50 μg of EN-Ctrl, EN-06, MO-Ctrl, or MO-06. The expression of the 4R-tau/3R-tau isoforms in the hippocampus were analyzed at 2, 6, 12, and 24 weeks post-injection by RT-PCR. A representative ENA-ASO gel image is shown in the left panel, and an MOE-ASO gel image is shown in the right panel. Band intensities were quantified and plotted (left graph - ENA-ASO; right graph - MOE-ASO). EN-Ctrl at 2 (n = 6), 6 (n = 6), 12 (n = 6), 24 (n = 6), 50 (n = 5), and 100 weeks (n = 5); EN-06 at 2 (n = 6), 6 (n = 6), 12 (n = 5), 24 (n = 6), 50 (n = 6), and 100 weeks (n = 6). MO-Ctrl at 2 (n = 6), 6 (n = 7), 12 (n = 6), and 24 weeks (n = 6); MO-06 at 2 (n = 6), 6 (n = 7), 12 (n = 6), and 24 weeks (n = 6). Statistical differences between EN-Ctrl and EN-06, and MO-Ctrl and MO-06 were assessed via two-way ANOVA with Bonferroni correction (***p < 0.001). Data are mean ± SD. (B) The same time course was evaluated by immunoblot (left: ENA-ASO; right: MOE-ASO). EN-Ctrl at 2 (n = 6), 6 (n = 7), 12 (n = 6), and 24 weeks (n = 6); EN-06 at 2 (n = 5), 6 (n = 5), 12 (n = 6), and 24 weeks (n = 6). MO-Ctrl at 2 (n = 5), 6 (n = 6), 12 weeks (n = 6), and 24 weeks (n = 6); MO-06 at 2 (n = 6), 6 (n = 8), 12 (n = 6), and 24 weeks (n = 6). Statistical differences were assessed via two-way ANOVA with Bonferroni correction (**p < 0.01). Data are mean ± SD.

of hippocampal dendritic spines. Similar phenotypic rescue was observed during late-stage disease with little evidence of abnormally phosphorylated tau or neurodegeneration of the hippocampus.

An increase in phosphorylated tau has been linked to elevated 4R-tau levels, for example through mutations in the *MAPT* gene, a mutation in *VCP*, or reduced interaction of FUS/SFPQ.[7,39–41] The amelioration of FTLD-like phenotypes, including tau phosphorylation, by EN-06 has primarily attributed to its exon 10-skipping effects, although minimal reductions in *MAPT* transcription may also have contributed. In addition, a decrease in 4R-tau would theoretically result in increased 3R-tau if total tau levels were preserved. However, this study found no significant increase in 3R-tau, particularly at the protein level. A slight reduction in total *MAPT* levels by EN-06 may explain this finding, and differences in the stability or degradation of 3R-tau vs. 4R-tau could further account for the discrepancy.

Generally, ASOs that regulate splicing exhibit high stability and have a long-lasting pharmacological effect as compared with RNase H1-dependent ASOs that contain a central stretch of unmodified DNA nucleotides (i.e., gapmer ASOs). Notably, a single ICV injection of a moderate amount (50 μg) of EN-06 elicited efficient and persistent skipping of *MAPT* exon 10 for as long as 2 years in the mouse brain. Consistent with this, LC-MS quantification of ASO tissue concentrations revealed a long half-life (∼6 months) for EN-06 in the mouse brain. For reference, the estimated half-life of nusinersen, an MOE-modified ASO drug that regulates target splicing in spinal muscular atrophy (SMA), was 71 days in the mouse brain following ICV injection.[42] This highlights the remarkable ability of ENA chemistries to regulate splicing in the central nervous system (CNS). This moiety can thus allow for a reduction in therapeutic dosage and dosing frequency, thereby minimizing any adverse off-target hybridization and class effects associated with ASO chemistries, as well as reducing the burden of repeated administrations.

Similar to MOE, a moderate dosage of ENA significantly curtailed the inflammation often observed with ASO chemistries, as the levels of *Aif1* and serum markers of liver and renal function were comparable to controls. Further, LC-MS quantification of tissue concentrations of

EN-06 revealed that the relative absence of liver and kidney toxicity was likely attributable to the shorter focal tissue half-life of EN-06. The enhanced efficacy and persistence of the ENA-modified ASO in regulating *MAPT* splicing than MOE-modified ASOs in FTLD model mice and human iPSC-derived neurons highlight the potential of EN-06 as a clinical therapeutic for 4R tauopathies.

While familial cases of FTLD characterized by *MAPT* gene mutations that promote 4R-tau accumulation represent clear opportunities for EN-06 intervention, the chemistry is also broadly applicable to sporadic cases of FTLD, PSP, and CBD. However, the diverse clinical presentations and lack of precise diagnostic tests for tauopathies may pose challenges to the therapeutic application of EN-06, especially at early disease stages. As such, the deployment of ASO-based intervention strategies would be aided by the development of laboratory- and imaging-based diagnostic biomarkers of 4R-tauopathies. The detection of tau isoforms in plasma extracellular vesicles shows strong potential as both a diagnostic and prognostic biomarker for 4R-tauopathies.[43]

In addition to the long-acting efficacy and safety of EN-06, our study demonstrated that EN-06 is largely targeted to the hippocampus, one of the primary regions affected by tauopathies. These properties suggest that RNA-splicing-targeted therapies that avoid affecting tau function in the CNS, even during the prodromal phase, may be viable intervention strategies. Further, recent advances in the PK/PD of ENA-modified ASOs, which surpass the potencies of gapmer ASOs, serve to increase drug-target engagement and advance treatment options for patients with cognitive impairment.

To further evaluate EN-06's safety, we plan to conduct additional pre-clinical (non-human) studies using a high dose of EN-06. These studies are expected to provide more robust data on EN-06's safety, efficacy, and feasibility in a system more closely resembling human physiology, which is necessary for initiating first-in-human trials.

MATERIALS AND METHODS

ENA-ASO

Literature reviews and prediction tools (ESE finder)[23] facilitated the design of 13 ASO sequences that annealed to sequences within exon 10 of *MAPT* or its flanking intron 9 or intron 10, and four non-specific control sequences. The nucleotide sequences of each ASO are shown in Table S2. Figure 1A shows the target regions of the ASOs, which were synthesized according to conventional methods. ENA was used for cytosine and thymine, and all phosphate groups were phosphorothioated. For comparative purposes, ASOs with MOE-modified sequences were also synthesized.

Assessment of tau splicing *(in vitro)*

Human cultured cells (HEK293 cells) were transfected with 50 nM of each ASO using Lipofectamine 2000 (#11668027, ThermoFisher Scientific, Waltham, MA, USA). Forty-eight hours after transfection, cultured cells were lysed using TRIzol (#NK-41596018, Thermo Fischer Scientific) and RNA was extracted using an RNeasy Mini Kit (#74104, Qiagen, Hilden, Germany). The extracted mRNA was reverse transcribed into cDNA using ImProm-II Reverse Transcriptase (#M314A, Promega, Madison, WI, USA), and the cDNA was then used as a template for semiquantitative RT-PCR. RT-PCR was performed using primers (forward: CCATGCCAGACCTGAAG AAT, sequence number 78; reverse: TGCTCAGGTCAACTGGT TTG, sequence number 79), AmpliTaq DNA Polymerase (#N8080152, ThermoFischer Scientific), and P-32 deoxycytidine 5′-triphosphate (#NEG013H, PerkinElmer, Waltham, MA, USA).

Mice and ASO injection

The TAU KO; hT-PAC-N (B6.Cg-*Mapt*^{tm1Hnd} Tg(MAPT)1Gds/Mmjax) mice were purchased from Jackson Laboratories (#017326). The mice produce normal human tau (*MAPT*) but no endogenous mouse tau (*Mapt*). Mice were maintained by backcrossing with Tau KO mice previously established by the Dawson Lab.[44] Mice were individually housed and kept on a 12-h light/dark schedule. Mice had free access to food and water. All mice used for experiments were male. To eliminate environmental effects on body weight, mice used for the experiments depicted in Figure 1 were kept in two different animal facilities, the Division of Experimental Animals, Nagoya University School of Medicine, and the Center for Animal Research and Education (CARE), Nagoya University. All animal experiments were performed in accordance with the National Institutes of Health Guide for the Care and Use of Laboratory Animals and were approved by the Nagoya University Animal Experiment Committee and the Shiga University of Medical Science Animal Care and Use Committee. All methods were carried out in accordance with relevant guidelines and regulations. All methods are reported based on the ARRIVE guidelines.

For ASO administration, 6-week-old mice were stereotaxically injected into the right ventricle with 5 μL of ASO (0–200 μg) in saline. A 10-μL syringe (1701RN, Hamilton, Reno, NV, USA) was used for the injection. The coordinates for injection were 0.2 mm posterior to the bregma suture, 1 mm lateral from the sagittal suture, and a depth of 2–3 mm. Following predetermined intervals (2, 6, 12, and 24 weeks), specimens from the cortex, hippocampus, liver, kidney, and serum were collected under anesthesia.

Assessment of tau splicing *(in vivo)*

RNA was extracted from the cortex and hippocampus using an RNeasy Lipid Tissue Mini Kit (#74804, Qiagen). The extracted mRNA was reverse transcribed into cDNA as described above. RT-PCR amplification was performed using Taq DNA Polymerase (#RR006A, Takara Bio, Otsu, Japan) and primers described in Table S3. PCR products were electrophoresed with 10× loading buffer (#9157, TaKaRa) and imaged with a UV camera. Detailed methods describing protein extraction from tissues and immunoblotting have been outlined previously.[7]

Quantification of EN-06 in tissues

ASOs were extracted from tissue homogenate samples by solid-phase extraction using Clarity OTX (Phenomenex, Torrance, CA, USA). An

internal standard was added at a final concentration of 0.5 μM prior to extraction.

A reversed phase ion-pair high-performance liquid chromatography-mass spectrometry (HPLC-MS) system consisting of Vanquish Horizon (ThermoFisher Scientific) and a mass spectrometer Orbitrap Exploris240 (ThermoFisher Scientific) was used to quantify ASOs. Tissue homogenate extracts were reconstituted in distilled water containing 20% methanol, and then injected into an ACQUITY UPLC Oligonucleotide BEH C18 column (2.1 × 50 mm, 1.7 μm particles; Waters, Milford, MA, USA). The column was maintained at 55°C and a flow rate of 0.3 mL/min. Solvent A consisted of 0.1% (7.2 mM) triethylamine (TEA) and 0.6% (57 mM) 1,1,1,3,3,3-hexafluoro-2-propanol (HFIP) in H_2O (v/v/v). Solvent B consisted of 0.1% TEA and 0.6% HFIP in methanol (v/v/v). Solvent B was maintained at 10% for 0.5 min, then increased to 50% in 4.5 min. Mass spectrometry was performed using the high-resolution selected ion monitoring mode.

Blood biochemistry analysis

A biochemical panel of blood analytes was determined using a 100-μL aliquot of mouse sera. An automated biochemical analyzer (VetScan VS2, Abaxis, Union City, CA, USA) equipped with a multirotor II VCP (Abaxis) was used to measure the levels of aspartate transaminase, alanine aminotransferase, total bilirubin, creatinine, and blood urea nitrogen.

Stereotactic injection of AAV into the hippocampus

To establish an FUS knockdown hTau mouse model, we injected 1 μL of AAV-S1/shRNA-FUS or -control virus (8.0 × 10^{10} VG) into the bilateral hippocampus of hTau mice. shRNAs used in this study are described in Table S3. AAVs (8.0 × 10^{10} VG of AAV per 1 μL solution) were bilaterally injected into the hippocampus (1.58 mm posterior to bregma, 1.50 mm lateral to midline, 1.80 mm below the skull surface) of 6-week-old male hT-PAC-N mice at a flow rate of 0.5 μL/min. After injection, mice were allowed to recover for 6 weeks. A detailed methodological description of AAV generation has been described previously.[7]

Behavioral analysis

Behavioral assays were conducted on mice aged 12–14 weeks. Mice were evaluated in an elevated-plus maze test and an open field test using the same experimental settings as those described previously.[27]

The feeding behavior patterns were analyzed by quantifying impaired feeding states in terms of spatial feeding behavior patterns defined as "fixated feeding" (i.e., a preference for a particular bait location) as described previously.[28]

Golgi-Cox staining

The brain section was prepared using a GolgiStain kit (FD Neuro Technologies, Ellicott City, MD, USA) with total and mature spine morphology analyzed as described previously.[27]

Immunofluorescent study

To assess the tissue distribution of EN-06, tissues were fixed in 4% PFA for a week and then paraffin-embedded. For antigen retrieval, paraffin-embedded sections were treated with 0.2 M HCl for 10 min. A custom mouse anti-EN-06 IgG antibody was generated as described previously.[45] The sections were stained with an anti-EN-06 antibody (1:500) overnight followed by secondary antibody incubation for 1 h.

To quantify tau phosphorylation in the brains of hTau mice, paraffin-embedded sections were treated with citrate buffer and then boiled. The PHF1 antibody was a gift from Dr. Peter Davies as described previously.[7] Immunofluorescent analyses were performed as described previously.[7,46] Images were taken using a BZ-X700 microscope (Keyence, Osaka, Japan) and the associated software was used to quantify the signals and signal-positive regions.

Comparison of ENA-ASO with MOE-ENA

We compared the *in vitro* efficiency of tau splicing among four ENA-modified ASOs (EN-Ctrl, EN-03, EN-05, and EN-06) and four MOE-modified ASOs (MO-Ctrl, MO-03, MO-05, and MO-06) targeting the same sequences. We also compared their *in vivo* efficiency using EN-Ctrl, EN-06, MO-Ctrl, and MO-06.

Administration of ENA-ASOs to human-induced pluripotent stem cell (iPSC)-derived neurons

The 201B7 hiPSCs were kindly provided by Dr. Yamanaka at Kyoto University.[47] Maintenance and differentiation of the hiPSCs were as described previously.[48,49] The precise culture and differentiation conditions are described elsewhere.[7] Four days after adherent differentiation, the cells were infected with a lentivirus encoding shRNA targeting human FUS (i.e., sh-hFUS1) as described previously.[7] ASOs to promote skipping of *MAPT* exon 10 (EN-06) or a control ASO (EN-Ctrl) were transfected with Lipofectamine 2000 (ThermoFisher Scientific) at concentrations of 20 nM or 40 nM at 7 days post-differentiation. Half of the medium was changed every 2–3 days. Four weeks after the adherent differentiation, the cells were harvested for analysis. The hiPSC experimental procedures were approved by the Aichi Medical University School of Medicine ethics committee (approval number 14-004, 2020-213, and 2022-431).

Statistical analysis

Statistical analyses were performed using JMP 17 (SAS Institute, Drive Cary, NC, USA) and GraphPad Prism (GraphPad Software, Inc, San Diego, CA, USA). The Anderson-Darling test was used to test the normality of samples in groups. For the statistical analysis of the two groups, Welch's t test was used as described in the figure legend. In experiments with more than two groups, one-way ANOVA post hoc Tukey's or Dunnett's comparison tests were used. ENA-ASO and MOE-ASO data were analyzed by two-way ANOVA followed by Bonferroni correction. The data are expressed as mean ± SD. All statistical tests were two-sided.

DATA AVAILABILITY

The datasets used and/or analyzed during the current study are available from the corresponding author upon reasonable request.

ACKNOWLEDGMENTS

The authors are grateful to the CARE (The Center for Animal Research and Education) at Nagoya University, the Division of Experimental Animals at Nagoya University School of Medicine, and the Research Center for Animal Life Science, Shiga University of Medical Science for technical support with the animal experiments. All experimental procedures involving the use of hiPSCs were approved by the ethics committee of the Aichi Medical University School of Medicine. This research was supported by AMED under Grant Number JP24ym0126113 (S.I.), JP20dm0107059 (G.S.), JP22bm0804020 (Y.O.), and JP24bm1423003 (Y.O.). This work was supported in part by JSPS KAKENHI; JP23K21410 (S.I.). It was also partially supported by the Research Support Project for Life Science and Drug Discovery (Basis for Supporting Innovative Drug Discovery and Life Science Research [BINDS]) from AMED under Grant Number JP24ama121053 (K. Kanamitsu).

AUTHOR CONTRIBUTIONS

Conceptualization, K.I.-E., K.S., G.S., and S.I. Mice experiments, K.I.-E., K. Kawai, Y.F., N.I., M.I., D.T., and S.I. ASO preparation, T.F. and M.N. AAV preparation, S.H. and H.O. Investigation, K.I.-E., K.S., K. Kawai, Y.F., and S.I. Quantification of EN-06, E.W., K. Kanamitsu, M.M., A.F.A., and S.I. Experiments using iPSC-derived neurons, Y.O. Data analysis, K.I.-E., K.S., Y.F., Y.O., E.W., K. Kanamitsu, M.K., H.W., and S.I. Writing – Original Draft, K.I.-E., K.S., G.S., and S.I. Writing – Review & Editing, G.S. and S.I. Funding Acquisition, G.S. and S.I.

DECLARATION OF INTERESTS

K.I.-E., K.S., T.F., M.N., S.I., and G.S. are listed as inventors on a patent titled "Oligonucleotides for Controlling Tau Splicing and Their Use" (JP Patent No. 73407994, China Patent No. ZL201980044932.7), which has been granted in Japan and China, and is currently under examination in the United States and the European Patent Office (EP). This patent covers the ENA-modified antisense oligonucleotide (EN-06) described in this paper. The authors may have financial interests in the commercialization of this technology. Y.O. is a scientific advisor of Kohjin Bio Co., Ltd.

SUPPLEMENTAL INFORMATION

Supplemental information can be found online at https://doi.org/10.1016/j.omtn.2025.102503.

REFERENCES

1. Hong, M., Zhukareva, V., Vogelsberg-Ragaglia, V., Wszolek, Z., Reed, L., Miller, B.I., Geschwind, D.H., Bird, T.D., McKeel, D., Goate, A., et al. (1998). Mutation-specific functional impairments in distinct tau isoforms of hereditary FTDP-17. Science *282*, 1914–1917.
2. Umeda, T., Yamashita, T., Kimura, T., Ohnishi, K., Takuma, H., Ozeki, T., Takashima, A., Tomiyama, T., and Mori, H. (2013). Neurodegenerative disorder FTDP-17-related tau intron 10 +16C –> T mutation increases tau exon 10 splicing and causes tauopathy in transgenic mice. Am. J. Pathol. *183*, 211–225.
3. Yoshida, M. (2006). Cellular tau pathology and immunohistochemical study of tau isoforms in sporadic tauopathies. Neuropathology *26*, 457–470.
4. VandeVrede, L., Ljubenkov, P.A., Rojas, J.C., Welch, A.E., and Boxer, A.L. (2020). Four-Repeat Tauopathies: Current Management and Future Treatments. Neurotherapeutics *17*, 1563–1581.
5. Olney, N.T., Spina, S., and Miller, B.L. (2017). Frontotemporal Dementia. Neurol. Clin. *35*, 339–374.
6. Cummings, J.L., Gonzalez, M.I., Pritchard, M.C., May, P.C., Toledo-Sherman, L.M., and Harris, G.A. (2023). The therapeutic landscape of tauopathies: challenges and prospects. Alzheimers Res. Ther. *15*, 168.
7. Ishigaki, S., Fujioka, Y., Okada, Y., Riku, Y., Udagawa, T., Honda, D., Yokoi, S., Endo, K., Ikenaka, K., Takagi, S., et al. (2017). Altered Tau Isoform Ratio Caused by Loss of FUS and SFPQ Function Leads to FTLD-like Phenotypes. Cell Rep. *18*, 1118–1131.
8. Ishigaki, S., Riku, Y., Fujioka, Y., Endo, K., Iwade, N., Kawai, K., Ishibashi, M., Yokoi, S., Katsuno, M., Watanabe, H., et al. (2020). Aberrant interaction between FUS and SFPQ in neurons in a wide range of FTLD spectrum diseases. Brain *143*, 2398–2405.
9. Riku, Y., Iwasaki, Y., Ishigaki, S., Akagi, A., Hasegawa, M., Nishioka, K., Li, Y., Riku, M., Ikeuchi, T., Fujioka, Y., et al. (2022). Motor neuron TDP-43 proteinopathy in progressive supranuclear palsy and corticobasal degeneration. Brain *145*, 2769–2784.
10. Ferrer, I., Legati, A., Garcia-Monco, J.C., Gomez-Beldarrain, M., Carmona, M., Blanco, R., Seeley, W.W., and Coppola, G. (2015). Familial behavioral variant frontotemporal dementia associated with astrocyte-predominant tauopathy. J. Neuropathol. Exp. Neurol. *74*, 370–379.
11. Schoch, K.M., DeVos, S.L., Miller, R.L., Chun, S.J., Norrbom, M., Wozniak, D.F., Dawson, H.N., Bennett, C.F., Rigo, F., and Miller, T.M. (2016). Increased 4R-Tau Induces Pathological Changes in a Human-Tau Mouse Model. Neuron *90*, 941–947.
12. Bunker, J.M., Wilson, L., Jordan, M.A., and Feinstein, S.C. (2004). Modulation of microtubule dynamics by tau in living cells: implications for development and neurodegeneration. Mol. Biol. Cell *15*, 2720–2728.
13. Hasegawa, M. (2006). Biochemistry and molecular biology of tauopathies. Neuropathology *26*, 484–490.
14. Stoothoff, W., Jones, P.B., Spires-Jones, T.L., Joyner, D., Chhabra, E., Bercury, K., Fan, Z., Xie, H., Bacskai, B., Edd, J., et al. (2009). Differential effect of three-repeat and four-repeat tau on mitochondrial axonal transport. J. Neurochem. *111*, 417–427.
15. Ishigaki, S., Masuda, A., Fujioka, Y., Iguchi, Y., Katsuno, M., Shibata, A., Urano, F., Sobue, G., and Ohno, K. (2012). Position-dependent FUS-RNA interactions regulate alternative splicing events and transcriptions. Sci. Rep. *2*, 529.
16. Ludolph, A.C., and Wurster, C.D. (2019). Therapeutic advances in SMA. Curr. Opin. Neurol. *32*, 777–781.
17. Paton, D.M. (2017). Nusinersen: antisense oligonucleotide to increase SMN protein production in spinal muscular atrophy. Drugs Today *53*, 327–337.
18. Sahashi, K., Hua, Y., Ling, K.K., Hung, G., Rigo, F., Horev, G., Katsuno, M., Sobue, G., Ko, C.P., Bennett, C.F., et al. (2012). TSUNAMI: an antisense method to phenocopy splicing-associated diseases in animals. Genes Dev. *26*, 1874–1884.
19. Sahashi, K., Ling, K.K., Hua, Y., Wilkinson, J.E., Nomakuchi, T., Rigo, F., Hung, G., Xu, D., Jiang, Y.P., Lin, R.Z., et al. (2013). Pathological impact of SMN2 mis-splicing in adult SMA mice. EMBO Mol. Med. *5*, 1586–1601.
20. Morita, K., Hasegawa, C., Kaneko, M., Tsutsumi, S., Sone, J., Ishikawa, T., Imanishi, T., and Koizumi, M. (2001). 2'-O,4'-C-ethylene-bridged nucleic acids (ENA) with nuclease-resistance and high affinity for RNA. Nucleic Acids Res. Suppl. *1*, 241–242.
21. Kimura, T., Whitcomb, D.J., Jo, J., Regan, P., Piers, T., Heo, S., Brown, C., Hashikawa, T., Murayama, M., Seok, H., et al. (2014). Microtubule-associated protein tau is essential for long-term depression in the hippocampus. Philos. Trans. R. Soc. Lond. B Biol. Sci. *369*, 20130144.
22. Oberrauch, S., Metha, J.A., Brian, M.L., Barnes, S.A., Featherby, T.J., Lawrence, A.J., Hoyer, D., Murawski, C., and Jacobson, L.H. (2021). Reward motivation and cognitive flexibility in tau null-mutation mice. Neurobiol. Aging *100*, 106–117.
23. Cartegni, L., Wang, J., Zhu, Z., Zhang, M.Q., and Krainer, A.R. (2003). ESEfinder: A web resource to identify exonic splicing enhancers. Nucleic Acids Res. *31*, 3568–3571.
24. Alhamadani, F., Zhang, K., Parikh, R., Wu, H., Rasmussen, T.P., Bahal, R., Zhong, X.B., and Manautou, J.E. (2022). Adverse Drug Reactions and Toxicity of the Food and Drug Administration-Approved Antisense Oligonucleotide Drugs. Drug Metab. Dispos. *50*, 879–887.
25. Hua, Y., Sahashi, K., Hung, G., Rigo, F., Passini, M.A., Bennett, C.F., and Krainer, A.R. (2010). Antisense correction of SMN2 splicing in the CNS rescues necrosis in a type III SMA mouse model. Genes Dev. *24*, 1634–1644.
26. Monine, M., Norris, D., Wang, Y., and Nestorov, I. (2021). A physiologically-based pharmacokinetic model to describe antisense oligonucleotide distribution after intrathecal administration. J. Pharmacokinet. Pharmacodyn. *48*, 639–654.
27. Udagawa, T., Fujioka, Y., Tanaka, M., Honda, D., Yokoi, S., Riku, Y., Ibi, D., Nagai, T., Yamada, K., Watanabe, H., et al. (2015). FUS regulates AMPA receptor function and FTLD/ALS-associated behaviour via GluA1 mRNA stabilization. Nat. Commun. *6*, 7098.

28. Fujioka, Y., Kawai, K., Endo, K., Ishibashi, M., Iwade, N., Tuerde, D., Kaibuchi, K., Yamashita, T., Yamanaka, A., Katsuno, M., et al. (2024). Stress-impaired reward pathway promotes distinct feeding behavior patterns. Front. Neurosci. *18*, 1349366.

29. Ahmed, R.M., Irish, M., Henning, E., Dermody, N., Bartley, L., Kiernan, M.C., Piguet, O., Farooqi, S., and Hodges, J.R. (2016). Assessment of Eating Behavior Disturbance and Associated Neural Networks in Frontotemporal Dementia. JAMA Neurol. *73*, 282–290.

30. Ahmed, R.M., Irish, M., Kam, J., van Keizerswaard, J., Bartley, L., Samaras, K., Hodges, J.R., and Piguet, O. (2014). Quantifying the eating abnormalities in frontotemporal dementia. JAMA Neurol. *71*, 1540–1546.

31. Ikeda, M., Brown, J., Holland, A.J., Fukuhara, R., and Hodges, J.R. (2002). Changes in appetite, food preference, and eating habits in frontotemporal dementia and Alzheimer's disease. J. Neurol. Neurosurg. Psychiatry *73*, 371–376.

32. Yokoi, S., Udagawa, T., Fujioka, Y., Honda, D., Okado, H., Watanabe, H., Katsuno, M., Ishigaki, S., and Sobue, G. (2017). 3'UTR Length-Dependent Control of SynGAP Isoform alpha2 mRNA by FUS and ELAV-like Proteins Promotes Dendritic Spine Maturation and Cognitive Function. Cell Rep. *20*, 3071–3084.

33. Polydoro, M., de Calignon, A., Suarez-Calvet, M., Sanchez, L., Kay, K.R., Nicholls, S.B., Roe, A.D., Pitstick, R., Carlson, G.A., Gomez-Isla, T., et al. (2013). Reversal of neurofibrillary tangles and tau-associated phenotype in the rTgTauEC model of early Alzheimer's disease. J. Neurosci. *33*, 13300–13311.

34. Santacruz, K., Lewis, J., Spires, T., Paulson, J., Kotilinek, L., Ingelsson, M., Guimaraes, A., DeTure, M., Ramsden, M., McGowan, E., et al. (2005). Tau suppression in a neurodegenerative mouse model improves memory function. Science *309*, 476–481.

35. DeVos, S.L., Miller, R.L., Schoch, K.M., Holmes, B.B., Kebodeaux, C.S., Wegener, A.J., Chen, G., Shen, T., Tran, H., Nichols, B., et al. (2017). Tau reduction prevents neuronal loss and reverses pathological tau deposition and seeding in mice with tauopathy. Sci. Transl. Med. *9*, eaag0481.

36. Zhang, D., Zhang, W., Ming, C., Gao, X., Yuan, H., Lin, X., Mao, X., Wang, C., Guo, X., Du, Y., et al. (2024). P-tau217 correlates with neurodegeneration in Alzheimer's disease, and targeting p-tau217 with immunotherapy ameliorates murine tauopathy. Neuron *112*, 1676–1693.e12.

37. Rovelet-Lecrux, A., Lecourtois, M., Thomas-Anterion, C., Le Ber, I., Brice, A., Frebourg, T., Hannequin, D., and Campion, D. (2009). Partial deletion of the MAPT gene: a novel mechanism of FTDP-17. Hum. Mutat. *30*, E591–E602.

38. Hoxha, V., Shrestha, G., Baloch, N., Collevechio, S., Laszczyk, R., and Roman, G. (2024). TBI and Tau Loss of Function Both Affect Naive Ethanol Sensitivity in Drosophila. Int. J. Mol. Sci. *25*, 3301.

39. Capano, L.S., Sato, C., Ficulle, E., Yu, A., Horie, K., Kwon, J.S., Burbach, K.F., Barthelemy, N.R., Fox, S.G., Karch, C.M., et al. (2022). Recapitulation of endogenous 4R tau expression and formation of insoluble tau in directly reprogrammed human neurons. Cell Stem Cell *29*, 918–932.e8.

40. Hung, C., and Patani, R. (2024). Elevated 4R tau contributes to endolysosomal dysfunction and neurodegeneration in VCP-related frontotemporal dementia. Brain *147*, 970–979.

41. Wobst, H.J., Denk, F., Oliver, P.L., Livieratos, A., Taylor, T.N., Knudsen, M.H., Bengoa-Vergniory, N., Bannerman, D., and Wade-Martins, R. (2017). Increased 4R tau expression and behavioural changes in a novel MAPT-N296H genomic mouse model of tauopathy. Sci. Rep. *7*, 43198.

42. Rigo, F., Chun, S.J., Norris, D.A., Hung, G., Lee, S., Matson, J., Fey, R.A., Gaus, H., Hua, Y., Grundy, J.S., et al. (2014). Pharmacology of a central nervous system delivered 2'-O-methoxyethyl-modified survival of motor neuron splicing oligonucleotide in mice and nonhuman primates. J. Pharmacol. Exp. Ther. *350*, 46–55.

43. Chatterjee, M., Ozdemir, S., Fritz, C., Mobius, W., Kleineidam, L., Mandelkow, E., Biernat, J., Dogdu, C., Peters, O., Cosma, N.C., et al. (2024). Plasma extracellular vesicle tau and TDP-43 as diagnostic biomarkers in FTD and ALS. Nat. Med. *30*, 1771–1783.

44. Dawson, H.N., Ferreira, A., Eyster, M.V., Ghoshal, N., Binder, L.I., and Vitek, M.P. (2001). Inhibition of neuronal maturation in primary hippocampal neurons from tau deficient mice. J. Cell Sci. *114*, 1179–1187.

45. Kobayashi, N., Katsumi, S., Imoto, K., Nakagawa, A., Miyagawa, S., Furumura, M., and Mori, T. (2001). Quantitation and visualization of ultraviolet-induced DNA damage using specific antibodies: application to pigment cell biology. Pigment. Cell Res. *14*, 94–102.

46. Endo, K., Ishigaki, S., Masamizu, Y., Fujioka, Y., Watakabe, A., Yamamori, T., Hatanaka, N., Nambu, A., Okado, H., Katsuno, M., et al. (2018). Silencing of FUS in the common marmoset (Callithrix jacchus) brain via stereotaxic injection of an adeno-associated virus encoding shRNA. Neurosci. Res. *130*, 56–64.

47. Takahashi, K., Tanabe, K., Ohnuki, M., Narita, M., Ichisaka, T., Tomoda, K., and Yamanaka, S. (2007). Induction of pluripotent stem cells from adult human fibroblasts by defined factors. Cell *131*, 861–872.

48. Onodera, K., Shimojo, D., Ishihara, Y., Yano, M., Miya, F., Banno, H., Kuzumaki, N., Ito, T., Okada, R., de Araujo Herculano, B., et al. (2020). Unveiling synapse pathology in spinal bulbar muscular atrophy by genome-wide transcriptome analysis of purified motor neurons derived from disease specific iPSCs. Mol. Brain *13*, 18.

49. Shimojo, D., Onodera, K., Doi-Torii, Y., Ishihara, Y., Hattori, C., Miwa, Y., Tanaka, S., Okada, R., Ohyama, M., Shoji, M., et al. (2015). Rapid, efficient, and simple motor neuron differentiation from human pluripotent stem cells. Mol. Brain *8*, 79.

OMTN, Volume ***36***

Supplemental information

Correcting tau isoform ratios with a long-acting antisense oligonucleotide alleviates 4R-tauopathy phenotypes

Kuniyuki Iwata-Endo, Kentaro Sahashi, Kaori Kawai, Yusuke Fujioka, Yohei Okada, Eri Watanabe, Nobuyuki Iwade, Minaka Ishibashi, Moniruzzaman Mohammad, Asraa Faris Aldoghachi, Dilina Tuerde, Tsuyoshi Fujiwara, Shinobu Hirai, Haruo Okado, Masahisa Katsuno, Hirohisa Watanabe, Kayoko Kanamitsu, Masahiro Neya, Shinsuke Ishigaki, and Gen Sobue

Supplemental Figure Legends

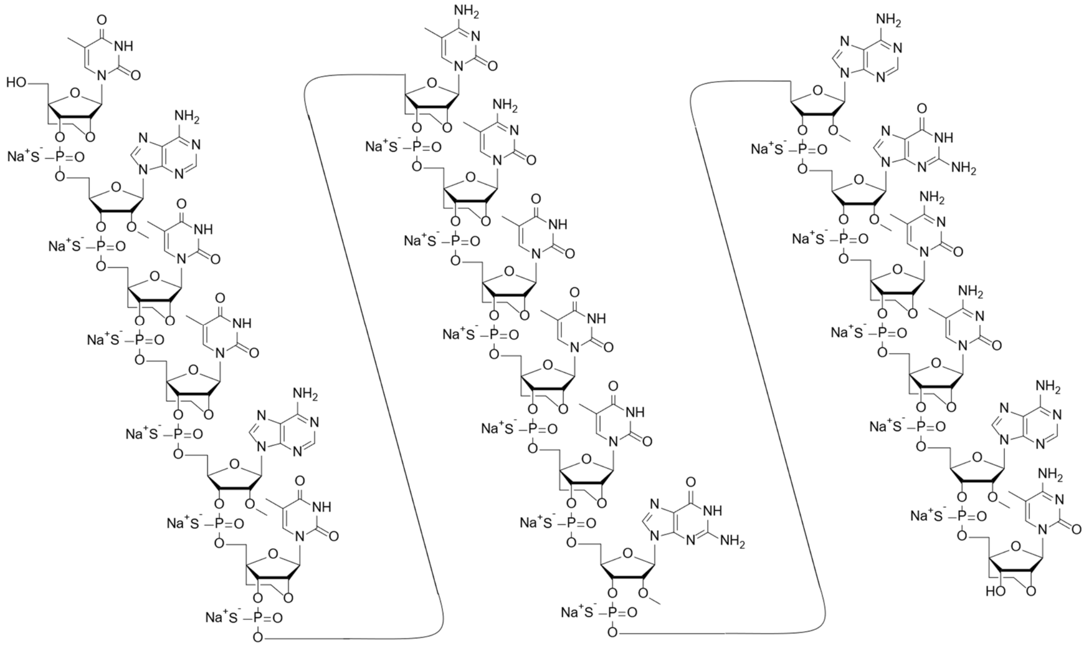

Figure S1. The structural formula of EN-06.

The structural formula of EN-06 sodium salt is shown.

Molecular Formula: $C_{210}H_{253}N_{59}O_{110}P_{17}S_{17}Na_{17}$

Molecular Weight: 6826.0370

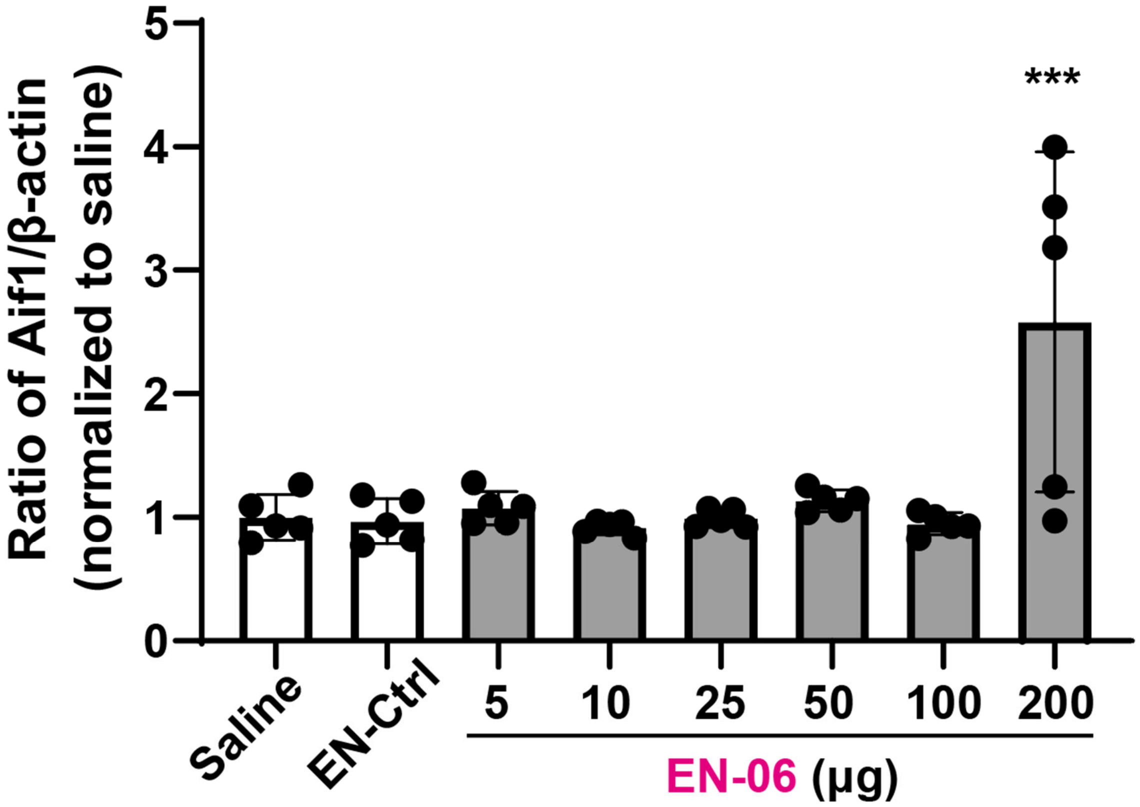

Figure S2. Inflammatory gene expression in the brain after a single ICV administration of EN-06.

The expression level of *Aif1*, which encodes the Iba1 protein, in brain homogenates from mice administered increasing doses (5, 10, 25, 50,100, and 200 μg) of EN-06 were assessed. The data shown represent the mean ± SD and are shown as *Aif1* per *β-actin* (n = 5 for each; one-way ANOVA; ***P < 0.001).

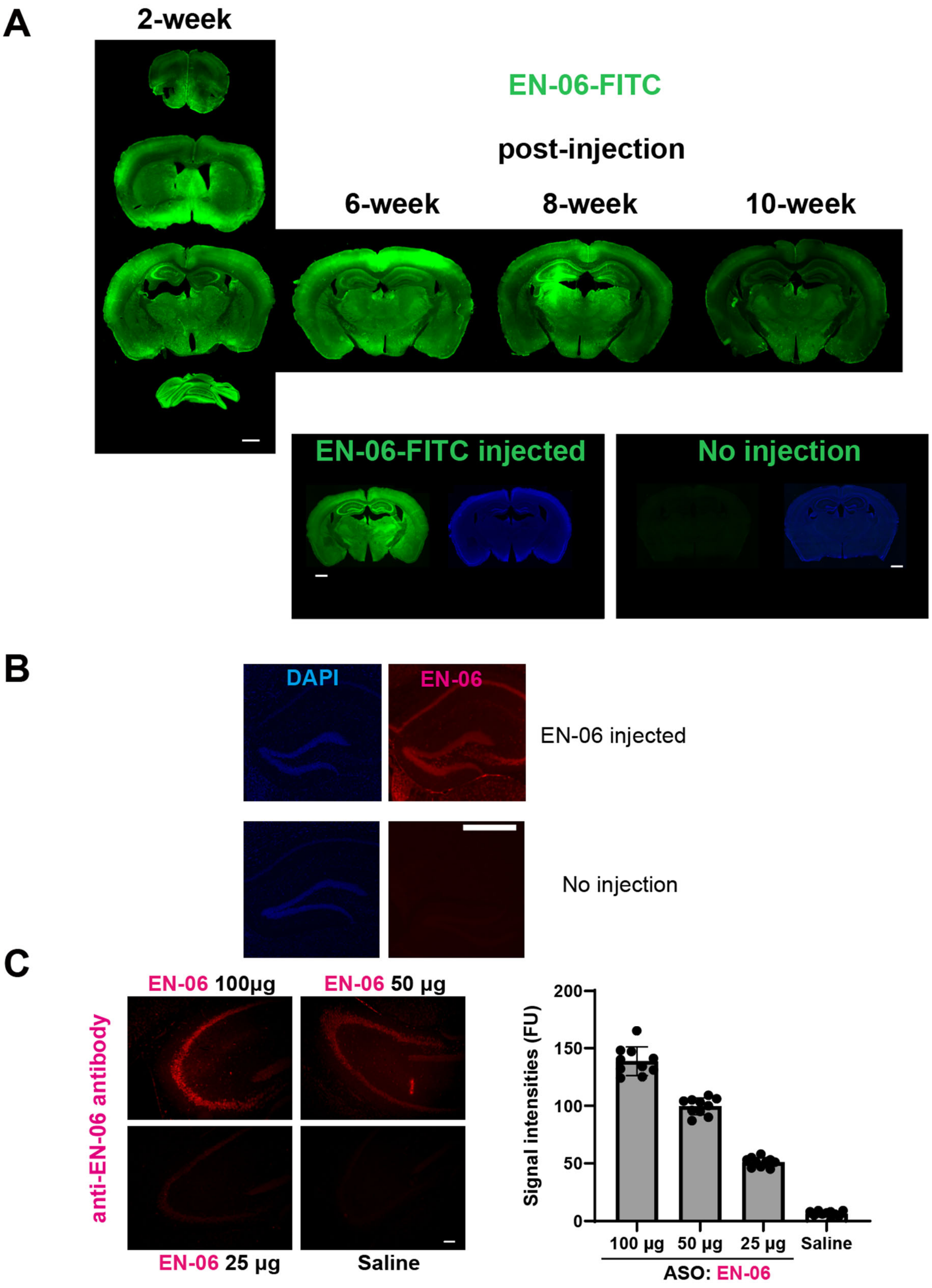
A
2-week
EN-06-FITC
post-injection
6-week
8-week
10-week
EN-06-FITC injected
No injection
B
DAPI
EN-06
EN-06 injected
No injection
C
EN-06 100µg
EN-06 50 µg
EN-06 25 µg
Saline
anti-EN-06 antibody
Signal intensities (FU)
200
150
100
50
0
100 µg
50 µg
25 µg
Saline
ASO: EN-06

Figure S3. Brain tissue distribution of ENA-ASOs targeting human *MAPT* exon 10.

(A) The brain distribution of FITC-labelled EN-06 at 2-, 6-, 8-, and 10 weeks post-ICV injection of 50 µg. A negative control section without injecting FITC-labelled EN-06 is shown with the section of FITC-labelled EN-06 at 6 weeks below. Scale bar = 1.0 mm. (B) Immunofluorescent study using a custom antibody against EN-06 in the brain. Mouse brain sections at 6 weeks post-ICV injection of 50 µg EN-06 were stained with an antibody against EN-06 (top). The negative control brain sections without EN-06 injection were stained with the same antibody (bottom). Scale bar = 0.5 mm. (C) Immunofluorescence imaging of hippocampal sections was done with a custom antibody against EN-06 after ICV injection of three different EN-06 doses (left panel). Scale bar = 100 µm. Signal intensities were quantified and plotted (right panel). The data shown represent the mean ± SD.

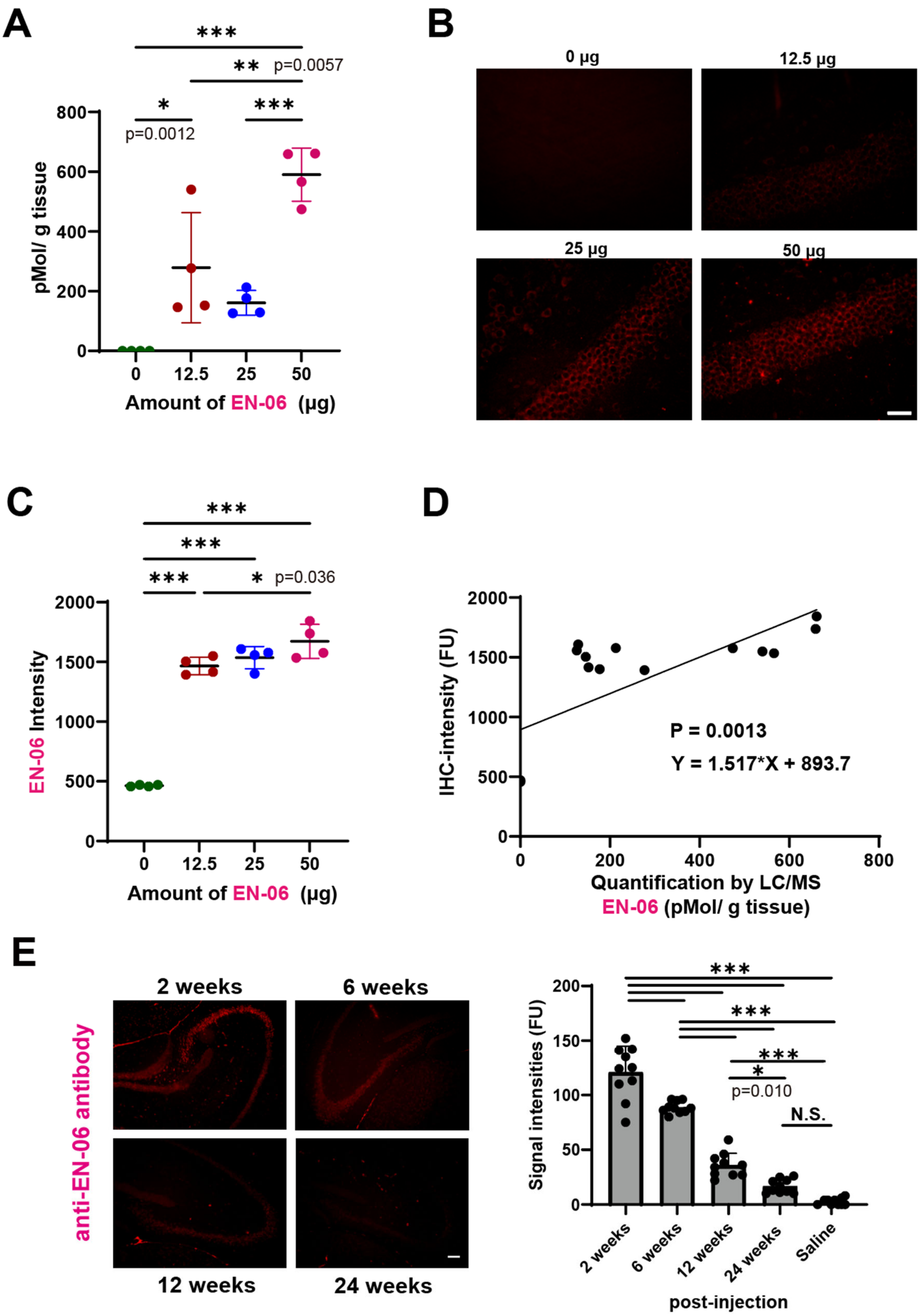
A
***
** p=0.0057
*
***
p=0.0012
pMol/ g tissue
800
600
400
200
0
0
12.5
25
50
Amount of EN-06 (µg)
B
0 µg
12.5 µg
25 µg
50 µg
C
***
***
***
* p=0.036
EN-06 Intensity
2000
1500
1000
500
0
0
12.5
25
50
Amount of EN-06 (µg)
D
IHC-intensity (FU)
2000
1500
1000
500
0
P = 0.0013
Y = 1.517*X + 893.7
0
200
400
600
800
Quantification by LC/MS
EN-06 (pMol/ g tissue)
E
anti-EN-06 antibody
2 weeks
6 weeks
12 weeks
24 weeks
Signal intensities (FU)
200
150
100
50
0
***
***
***
*
p=0.010
N.S.
2 weeks
6 weeks
12 weeks
24 weeks
Saline
post-injection

Figure S4. LC/MS-based and immunofluorescence-based quantification of EN-06 in the brain.

(A) The absolute tissue concentration of EN-06 was determined in the hippocampus of mice administered 4 doses (0, 12.5, 25, and 50 μg) of EN-06 by combining solid-phase extraction and LC/MS. The concentrations of EN-06 in the hippocampus of mice at 6 weeks post-injection were plotted and are shown as pMol/ g tissue (n = 4 for each dose; one-way ANOVA). (B) Immunofluorescent imaging was performed with a custom antibody against EN-06 using hippocampal sections of the mice shown in (A). Scale bar = 40 μm. (C) Signal intensities of the images in (B) were quantified and plotted. (D) Correlation of the absolute tissue concentrations of EN-06 as determined by LC/MS and immunofluorescence imaging (P = 0.0013). (E) Immunofluorescence imaging of hippocampal sections at various time points post-ICV injection of a 50-μg dose of EN-06 into 6-week-old hTau mice. Hippocampal sections were stained with a custom antibody against EN-06 (left panel). Scale bar = 100 μm. Signal intensities of the immunofluorescent images were quantified and plotted (right panel). *P < 0.05, **P < 0.01, ***P < 0.001.

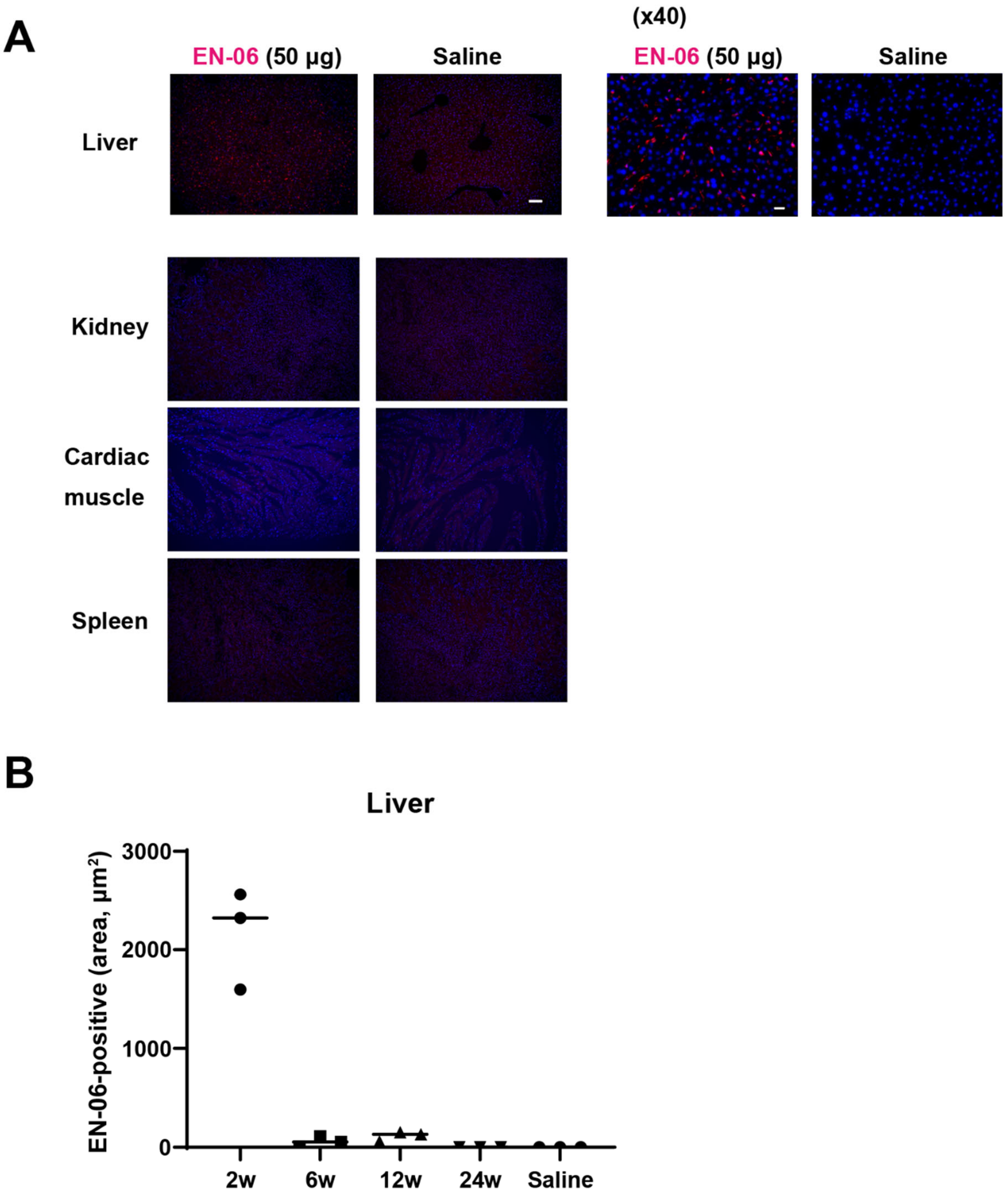

Figure S5. General tissue distribution of EN-06. (A) Immunofluorescent images of liver, kidney, cardiac muscle, and spleen sections of mice ICV injected 50 µg of EN-06 or saline. Sections were stained with a custom antibody against EN-06. Scale bar = 100 µm for left

images, 20 μm for right images. (B) The signal intensities in the liver sections were quantified and plotted (right panel). The data shown represent the mean ± SD (n = 3 for each).

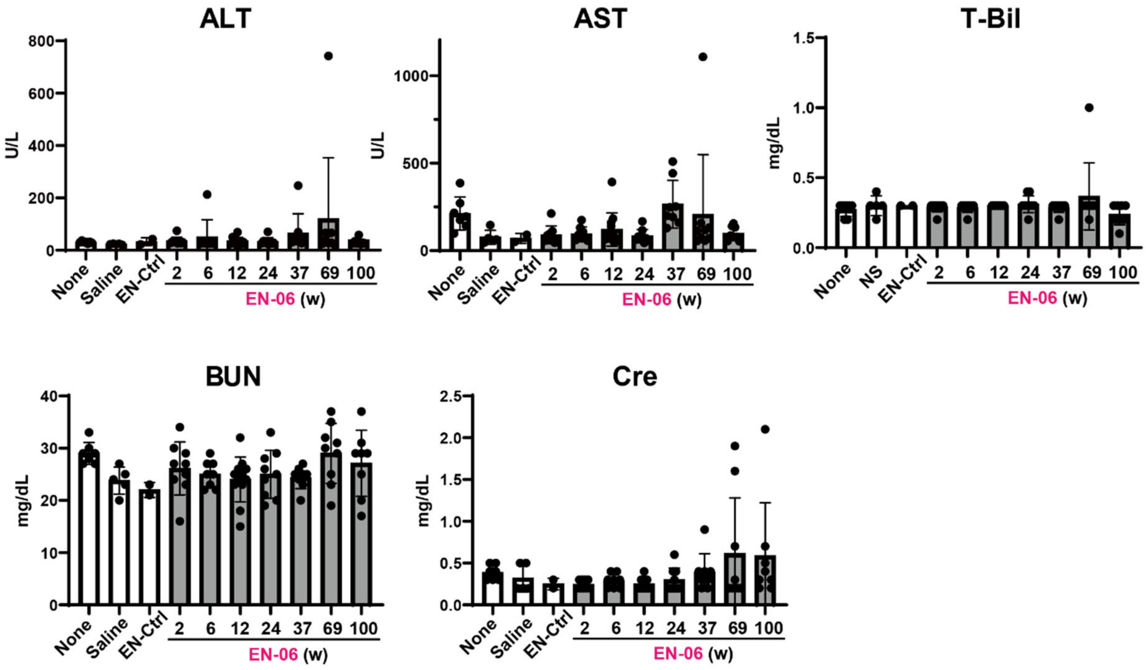

Figure S6. EN-06 has no significant negative effects on liver and renal function.

A panel of blood biochemical analytes associated with liver and renal function was assessed at various time points (2, 6, 12, 24, 37, 69, and 100 weeks) post-EN-06 administration. Mice administered the EN-Ctrl were assessed at 6 weeks, those administered the saline control at 2 weeks, and the non-injected control group was assessed immediately. Analytes measured included alanine aminotransferase (ALT), aspartate transaminase (AST), total bilirubin (T-Bil), blood urea nitrogen (BUN), and creatinine (Cre). There were no significant differences between the control group and any of the experimental groups (one-way ANOVA). The data shown represent the mean ± SD.

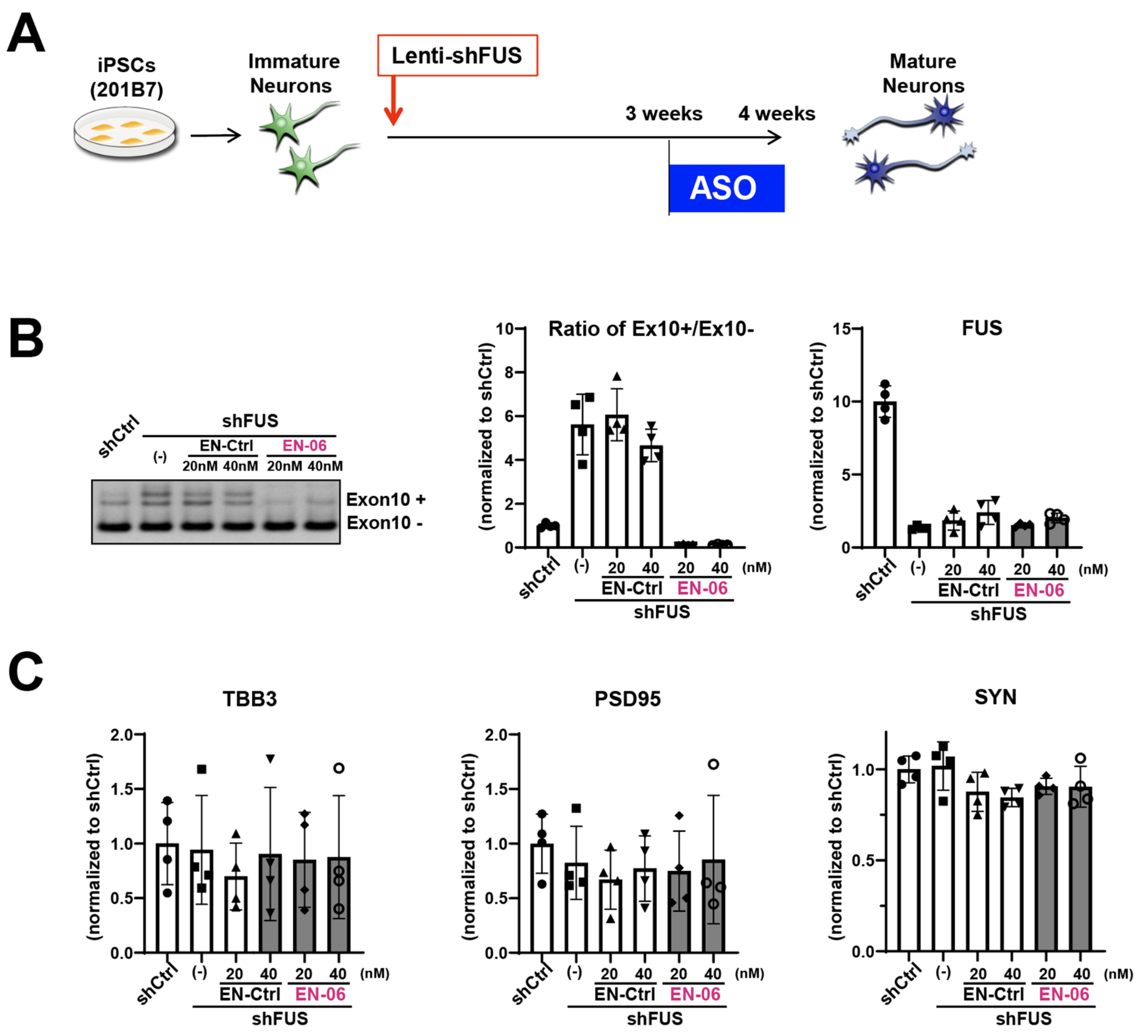

Figure S7. EN-06 restores the FUS-silencing induced increase in 4R-tau/3R-tau ratios in human-induced pluripotent stem cell (iPSC)-derived neurons. (A) Experimental scheme. Briefly, neurons infected with a lentivirus encoding shRNA were treated with 20 or 40 nM of each of the ASOs at 7 days post-differentiation. (B) Lentivirus-mediated shRNA targeting human FUS and a scramble control shRNA (shCtrl) were introduced into human iPSC-derived neurons. Neurons were then treated with 20 or 40 nM EN-06 or EN-Ctrl.

Alternative splicing of *MAPT* exon 10 was assessed by RT-PCR (left panel). The signals were quantified, and the isoform ratio was determined (middle graph). *FUS* expression was assessed by qRT-PCR (right graph). (C) Neuronal transcript levels of *βIII-tubulin* (TBB3), *PSD95*, and *synaptophysin* (SYN) were determined by qRT-PCR. The relative amount for each transcript was normalized to the housekeeping β-actin. The data shown represent the mean ± SD (n = 4 for each).

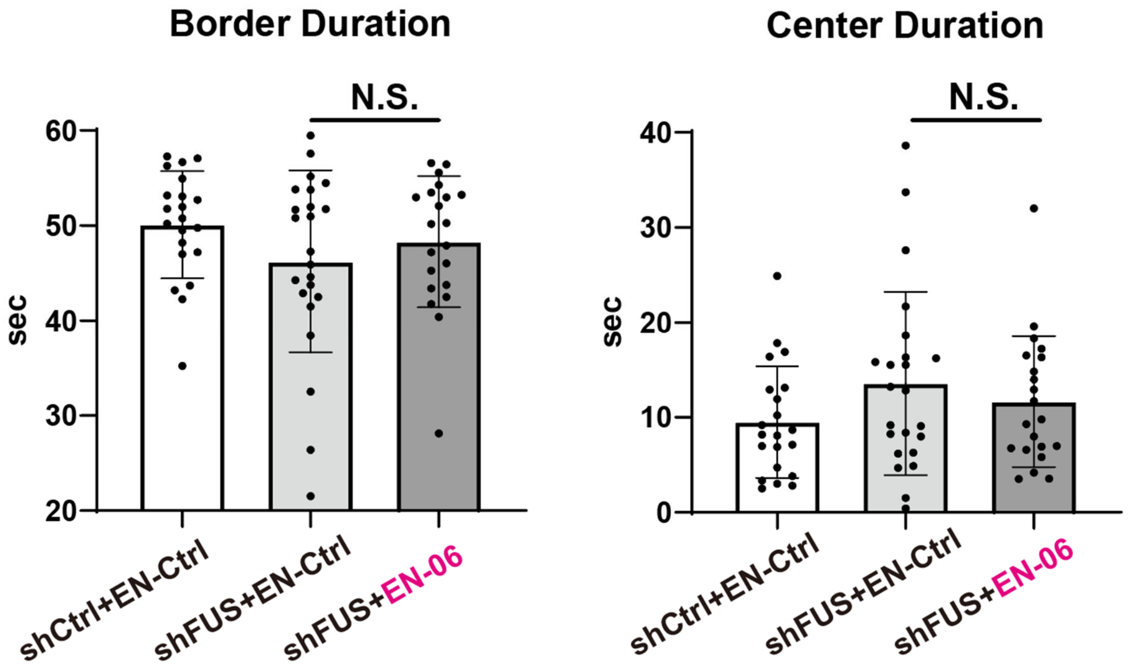

Figure S8. Open-field test behavioral assessment of the FUS knockdown hTau mouse model administered EN-06. Mice were treated with shCtrl + EN-Ctrl, shFUS + EN-Ctrl, or shFUS + EN-06. Time spent in the border (left graphs) and the center (middle graphs) area of an open field was determined (n = 21 for shCtrl + EN-Ctrl, n = 23 for shFUS +EN-Ctrl, and n = 21 for shFUS + EN-06; one-way ANOVA). N.S. denotes not significant. The data shown represent the mean ± SD.

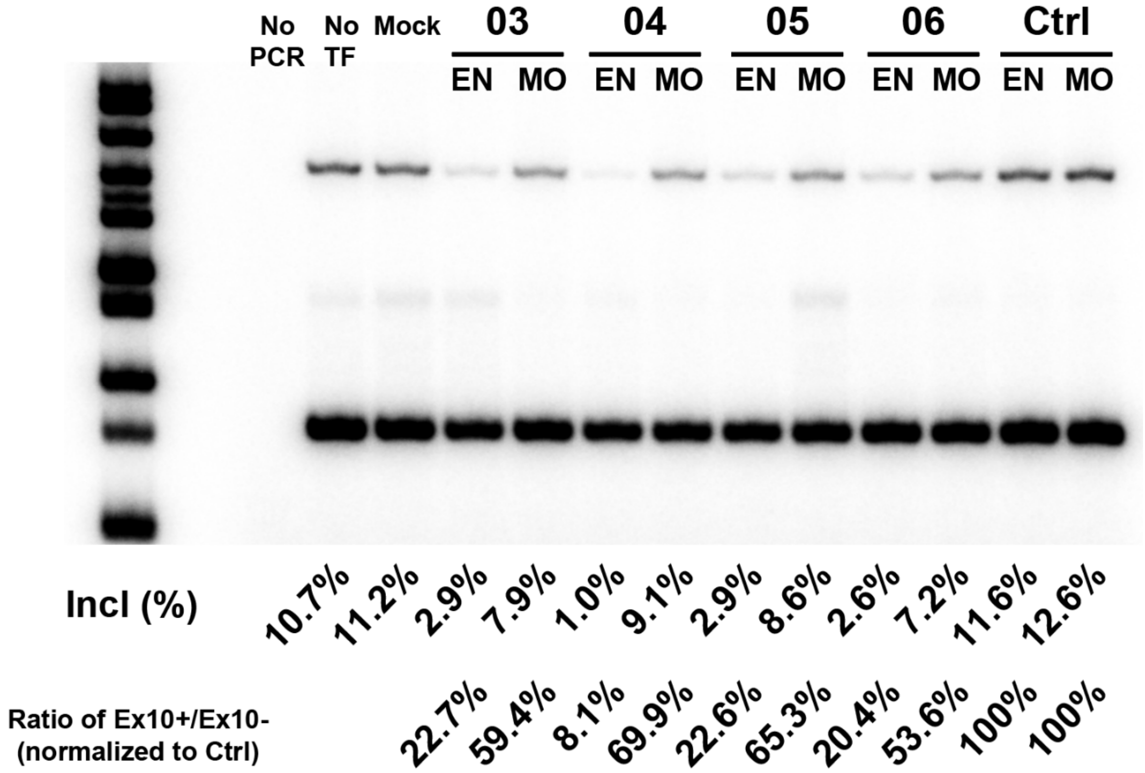

Figure S9. Efficiency of ENA- and MOE-modified ASOs targeting the same sequence to induce skipping of *MAPT* exon 10. RT-PCR was utilized to assess the effects of ENA- and MOE-modified ASOs (EN-03, MO-03, EN-04, MO-04, EN-05, MO-05, EN-06, and MO-06) and control ASOs (EN-Ctrl and MO-Ctrl) on the splicing of *MAPT* exon 10 in HEK293 cells. The percentage of exon 10-skipped transcripts was calculated relative to the total transcripts (sum of exon 10-skipped and exon 10-included transcripts) for each ASO. The ratio of exon 10-included to exon 10-skipped transcripts is shown, normalized to the ratio observed in the control, which was set to 100%.

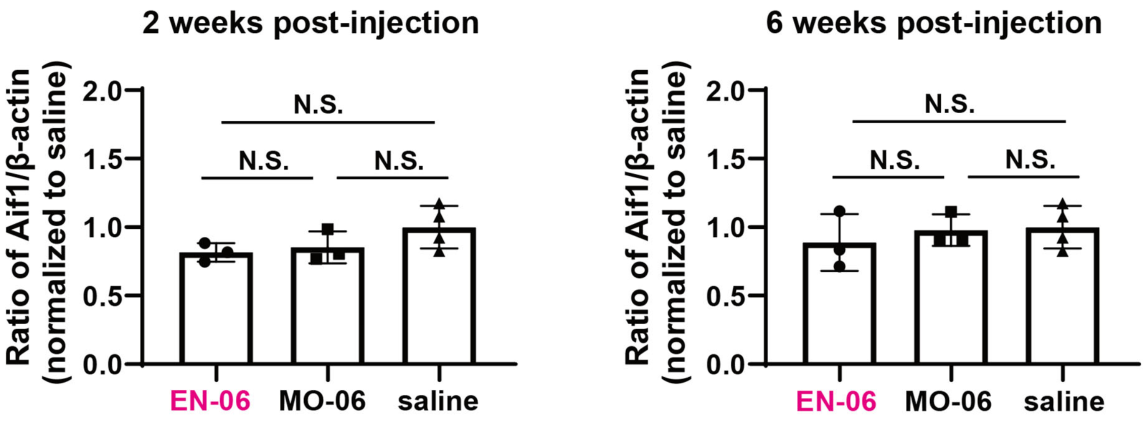

Figure S10. ***Aif1*** **expression in the brain after ICV administration of ENA- or MOE-modified ASO-06.**

The expression level of *Aif1*, which encodes the Iba1 protein, was assessed in brain homogenates of mice administered either EN-06 or MO-06. Data are shown as *Aif1* per *β-actin*; n = 3 for EN-06 and MO-06 and n = 4 for the saline control; one-way ANOVA). N.S. denotes not significant. The data shown represent the mean ± SD.

Table S1. ASOs used in this study.

Label	Sequence	Target	Function
EN-01	agCCagaaaaaaggaTga	Intron 9	Exon 10 skipping
EN-02	TggaCgTTgCTaagaTCC	Exon 10	Exon 10 skipping
EN-03	gCCaCaCTTggaCTggaC	Exon 10	Exon 10 skipping
EN-04	CCTTTgagCCaCaCTTgg	Exon 10	Exon 10 skipping
EN-05	TTaTCCTTTgagCCaCaC	Exon 10	Exon 10 skipping
EN-06	TaTTaTCCTTTgagCCaC	Exon 10	Exon 10 skipping
EN-07	gaTaTTaTCCTTTgagCC	Exon 10	Exon 10 skipping
EN-08	ggaCgTgTTTgaTaTTaT	Exon 10	Exon 10 skipping
EN-09	gCaTgggaCgTgTgaagg	Intron 10	Exon 10 inclusion
EN-10	gCaCggCgCaTgggaCgT	Intron 10	Exon 10 inclusion
EN-11	TTTaTTCTaTgCagTgTC	Intron 10	Exon 10 inclusion
EN-12	gCCCaagaaggaTTTaTT	Intron 10	Exon 10 inclusion
EN-13	ggCgCaTgggaCgTgTga	Intron 10	Exon 10 inclusion
EN-C1	CaTCTaagCaaCaaTTga	Non-specific	Control ASO
EN-C2 (EN-Ctrl)	CTCTTgaCgCaCaTCTgg	Non-specific	Control ASO
EN-C3	TTCCCTgaaggTTCCTCC	Non-specific	Control ASO
EN-C4	TCagTaaaCTTgaCaCCa	Non-specific	Control ASO

Uppercase letters: ENA-modified nucleotides

Lowercase letters: 2′-*O*-methylation-modified nucleotides

Table S2. Primers used for RT-PCR.

Gene	Forward	Reverse
mouse Fus	GGCTACTCCCAACAGAGCAG	GCTGTTTTGGGTCTGTCCAT
mouse β-actin	GCAAGTGCTTCTAGGCGGAC	AAGAAAGGGTGTAAAACGCAGC
mouse Aif1	AAGAGAGGCTGGAGGGGATC	GCTTCAAGTTTGGACGGCAG
human MAPT (exon10)	CCATGCCAGACCTGAAGAAT	TGCTCAGGTCAACTGGTTTG
human MAPT (whole)	GCAACATCCATCATAAACCAGGA	AGGGACCCAATCTTCGACTG
human FUS	AGCTCCCAATCGTCTTACGG	TTGCTGCTGTCCACCATAGC
human β-actin	GATCAAGATCATTGCTCCTCCT	GGGTGTAACGCAACTAAGTCA
human βIII-Tubulin	CATTCTGGTGGACCTGGAAC	ATACTCCTCACGCACCTTGC
human PSD95	ATATGTGAACGGGACCGAGG	TCACCGATGTGTGGGTTGTC
human SYN	GCCAACAAGACCGAGAGTGA	GAATTCGGCTGACGAGGAGT

Table S3. shRNAs used in this study.

shRNA Name	Target Sequence
FUS	GCAACAAAGCTACGGACAA
Ctrl	AATTCTCCGAACGTGTCACGT
